# Present and Future Options for Pharmacotherapy in Cardiovascular Disease: Hemodynamic and Mechanistic Therapeutic Targets

**DOI:** 10.3390/medsci14020331

**Published:** 2026-06-18

**Authors:** Francesc Cabré, Marta Cascante

**Affiliations:** 1 Department of Biochemistry & Molecular Biomedicine, University of Barcelona, 08028 Barcelona, Spain; fcabre@ub.edu; 2 Medical Area, Menarini Group, 08918 Badalona, Spain; 3 Biomedicine Institute, University of Barcelona, 08028 Barcelona, Spain; 4 CIBER of Hepatic and Digestive Diseases (CIBERHD), Institute of Health Carlos III (ISCIII), 28029 Madrid, Spain

**Keywords:** cardiovascular disease, cardiovascular pharmacology, hypertension, arrythmia, heart failure, cardiac hypertrophy

## Abstract

Cardiovascular diseases (CVDs) remain the leading global cause of morbidity and mortality, imposing an increasing clinical and socioeconomic burden. Despite significant therapeutic advances, optimal control of risk factors and long-term outcomes remain challenging, particularly in patients with complex comorbidities. This narrative review provides a comprehensive and up-to-date synthesis of pharmacological options across major cardiovascular domains, with a specific focus on hypertension, heart failure, arrhythmias, and hypertrophic cardiomyopathy, conditions in which hemodynamic, neurohormonal, and electrophysiological pathways play central roles. We summarize mechanisms of action, clinical evidence, safety profiles, and guideline-based indications of established therapies, highlighting their relevance to vascular tone regulation, neurohormonal modulation, endothelial signaling, and myocardial function, the mechanistic axes that intersect with pathways implicated in pulmonary vascular disease (PVD). In addition, we discuss emerging therapeutic targets and innovative agents such as renin-angiotensin-aldosterone system silencers, endothelin pathway modulators, SGLT2 inhibitors, soluble guanylate cyclase stimulators, myosin inhibitors, and other mechanism-based approaches. Current challenges and unmet clinical needs are examined in the context of translational relevance for PVD and the broader goal of advancing individualized pharmacotherapy. Continued therapeutic innovation targeting shared vascular, metabolic, and neurohormonal pathways holds promise for improving outcomes across both systemic and pulmonary vascular diseases.

## 1. Introduction

Cardiovascular diseases (CVDs) comprise heterogeneous disorders driven by abnormalities in vascular tone, neurohormonal activation, cardiac electrophysiology and myocardial structure, which collectively contribute to adverse outcomes across hypertension, heart failure, arrhythmias, and cardiomyopathies [1,2,3]. Dysregulation of the renin–angiotensin–aldosterone system (RAAS), endothelial dysfunction, impaired nitric oxide-soluble guanylate cyclase (NO–sGC) signaling, endothelin activation, sympathetic overactivity and calcium-dependent vasomotor tone are central mechanisms implicated in these conditions [4,5,6,7,8]. These pathways also show substantial overlap with those involved in pulmonary vascular disease (PVD), where aberrant vasoconstriction, vascular remodeling, inflammation, metabolic stress and right ventricular overload constitute major drivers of disease progression [9,10].

Recent therapeutic advances targeting neurohormonal signaling, myocardial energetics, soluble guanylate cyclase (sGC) activation, sodium–glucose cotransporter 2 (SGLT2)-mediated pathways and electrophysiological disturbances have expanded the pharmacological landscape for systemic CVD [11,12]. Several of these developments, particularly RAAS inhibition, endothelin pathway modulation, NO–sGC enhancement, sympathetic regulation and myosin-targeting therapies, bear translational relevance for PVD, where similar dysregulated signaling cascades contribute to increased pulmonary vascular resistance and right ventricular dysfunction [13,14,15].

We provide an updated synthesis of established and emerging pharmacotherapies for hypertension, heart failure, arrhythmias and hypertrophic cardiomyopathy. Emphasis is placed on their mechanistic underpinnings and on hemodynamic, neurohormonal and electrophysiological pathways that intersect with those implicated in PVD. By focusing on shared biological axes rather than disease-specific phenotypes, we highlight therapeutic insights with potential cross-disease applicability in systemic and pulmonary vascular disorders.

**Figure 1:**
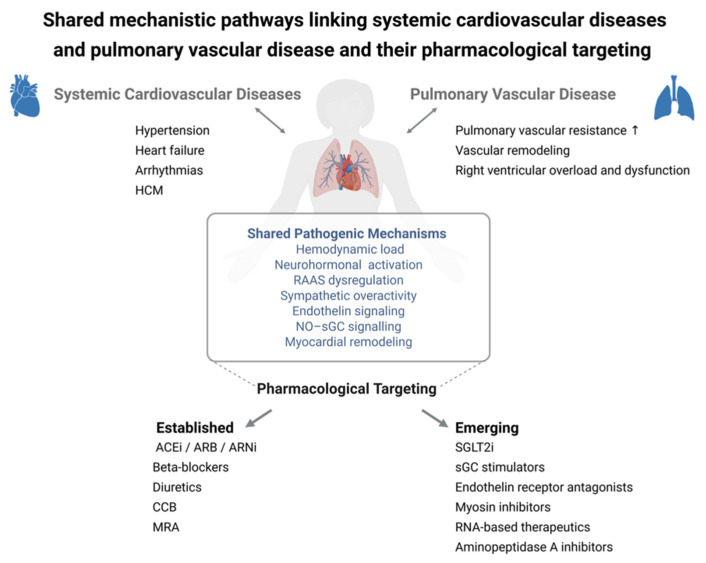
Shared mechanistic pathways linking systemic cardiovascular diseases and pulmonary vascular disease and their pharmacological targeting. Key pathophysiological processes, including hemodynamic load, neurohormonal activation, RAAS dysregulation, sympathetic overactivity, endothelin signaling, NO–sGC signaling, and myocardial remodeling, are common to both systemic and pulmonary vascular disorders. These interconnected mechanisms provide a conceptual framework for pharmacological intervention. Established therapies primarily target downstream pathways and remain the cornerstone of clinical management, whereas emerging mechanism-based approaches aim to modulate upstream processes, supporting the development of more integrated and translational therapeutic strategies across vascular territories. Created in BioRender: https://BioRender.com/99qyxkd (accessed on 1 May 2026). ACEi: angiotensin-converting enzyme inhibitors; ARB: angiotensin II receptor blocker; ARNi: angiotensin receptor–neprilysin inhibitor; CCB: calcium channel blocker; HCM: hypertrophic cardiomyopathy; MRA: mineralocorticoid receptor antagonist; NO–sGC: Nitric oxide-soluble guanylate cyclase; RAAS: renin–angiotensin–aldosterone system; sGC: soluble guanylate cyclase; SGLT2i: sodium–glucose cotransporter 2 inhibitor.

Figure 1 summarizes the shared mechanistic pathways linking systemic cardiovascular diseases and pulmonary vascular disease and illustrates how these convergent processes provide a common framework for established and emerging pharmacological strategies.

This narrative review is based on a comprehensive and critical appraisal of the most relevant and up-to-date literature available in the field. The selection of sources primarily includes articles published in recent years in peer-reviewed, indexed journals, with a particular emphasis on databases such as PubMed. In addition, current clinical practice guidelines, authoritative pharmacology textbooks/manuals and updated drug prescribing information (including official summary of product characteristics) have been systematically consulted. Priority was given to high-quality evidence, landmark clinical trials, and consensus recommendations to ensure an accurate and clinically meaningful synthesis of contemporary pharmacotherapeutic strategies.

This review aims to provide a comprehensive pharmacological overview as well as a translational description of the mechanisms involved in the described pathologies and the drugs used in their treatments. It is intentionally designed to address a broad biomedical audience that extends beyond the clinical cardiology setting. In particular, it aims to provide researchers in basic and translational biomedicine with a comprehensive and accessible overview of major cardiovascular pathologies and their current pharmacological management. Rather than focusing exclusively on clinical decision-making or highly specialized therapeutic algorithms, the emphasis is placed on integrating mechanistic insights with therapeutic strategies, thereby facilitating a deeper understanding of the molecular and pathophysiological processes underlying these diseases.

By systematically linking pharmacological interventions to their hemodynamic, neurohormonal, and cellular targets, this review seeks to bridge the gap between fundamental research and clinical application, offering a conceptual framework that may support both experimental investigation and interdisciplinary knowledge transfer, including its relevance to related conditions such as pulmonary vascular disease.

## 2. Risk Factors in Shared Mechanistic Pathways Between Systemic Cardiovascular Disorders and Pulmonary Vascular Disease

Several core biological pathways that drive systemic CVD are also central to the pathophysiology of PVD, particularly those regulating vascular tone, neurohormonal activation, inflammation, endothelial function and myocardial stress. Despite differences in clinical presentation, many of these conditions share common upstream mechanisms, notably dysregulation of RAAS, sympathetic overactivation, endothelin-mediated vasoconstriction, NO–sGC pathway impairment, and calcium-dependent vascular smooth muscle contraction [15,16,17].

RAAS overactivity contributes to increased vascular resistance, endothelial dysfunction and maladaptive remodeling in systemic hypertension. Similar alterations in angiotensin II and aldosterone signaling have been implicated in increased pulmonary vascular tone and right ventricular load. Endothelin-1, a potent vasoconstrictor elevated in resistant hypertension and heart failure, also plays a pivotal role in pulmonary vasoconstriction and vascular remodeling. Its dual role across vascular beds highlights mechanistic intersections that may inform therapeutic repurposing [8].

Impairment of the NO–sGC pathway, long recognized as a contributor to endothelial dysfunction in heart failure and hypertension, is likewise a hallmark of PVD, where reduced nitric oxide bioavailability and diminished sGC activity promote vasoconstriction and proliferative remodeling [15].

Altered cardiac loading conditions in systemic hypertension and heart failure—especially increased afterload, ventricular wall stress, and neurohormonal activation—share mechanistic continuity with right ventricular pressure overload in PVD, contributing to metabolic stress and maladaptive myocardial remodeling. Accumulating evidence indicates that systemic hemodynamic and neurohormonal pathways often operate in parallel within both circulations, creating opportunities for cross-disease therapeutic translation. From a translational perspective, these shared risk factors and underlying mechanisms may also contribute to pulmonary vascular disease, although their direct clinical impact in this context remains incompletely defined.

## 3. Antihypertensive Agents

Hypertension represents a major hemodynamic contributor to systemic CVD and shares several mechanistic pathways with pulmonary vascular dysfunction, particularly those involving RAAS activation, sympathetic tone, endothelial function and vascular remodeling. This condition, in which blood pressure (BP) in arteries is persistently elevated, remains one of the most prevalent and consequential cardiovascular (CV) risk factors globally, often necessitating combined lifestyle and pharmacological interventions to achieve optimal BP control.

Current international guidelines consistently endorse five primary antihypertensive drug classes: angiotensin-converting enzyme inhibitors (ACEi), angiotensin II receptor blockers (ARBs), beta-blockers (BBs), calcium channel blockers (CCBs), and diuretics, as the foundation of pharmacotherapy (Table 1). This recommendation is supported by extensive evidence demonstrating their capacity to lower BP, reduce major CV events in placebo-controlled randomized trials, and provide broadly equivalent reductions in cardiovascular morbidity and mortality, indicating that treatment benefits arise primarily from BP reduction [18,19,20,21].

A comprehensive 2025 meta-analysis, comprising 484 randomized controlled trials and over 100,000 participants, reinforced the robust antihypertensive efficacy of these five drug classes and validated the predictable BP-lowering effects of both monotherapy and combination regimens. Furthermore, comparative effectiveness analyses have revealed clinically relevant differences in specific outcomes: a large real-world cohort study reported higher rates of adverse cardiac events among patients initiated on beta-blockers compared with those treated first-line with thiazide diuretics, while ACEi/ARBs and CCBs demonstrated similar CV risk profiles. These data highlight the importance of individualized drug selection based on comorbidities, age, and overall CV risk [22,23].

Despite these considerations, authoritative reviews continue to affirm the centrality of these five classes as the therapeutic backbone, supported by strong evidence for efficacy, safety, and outcome improvement. Additional antihypertensive agents, such as alpha-blockers and mineralocorticoid receptor antagonists (MRAs), play a more selective role due to either limited event-driven randomized clinical trial (RCT) evidence or a higher incidence of adverse effects. Nevertheless, these agents remain important adjuncts for patients whose BP is inadequately controlled despite optimized use of first-line therapy combinations [24]. Emerging literature further underscores pharmacologic innovation in hypertension management, including the development of agents targeting hypertension-mediated organ damage or metabolic disturbances, as summarized in recent therapeutic overviews. However, these new approaches complement rather than replace the established standard of care built upon the five foundational antihypertensive classes [25].

In the following subsections, established antihypertensive drug classes are reviewed with emphasis on their mechanisms, efficacy and safety.

### 3.1. Diuretics

Among first-line antihypertensive options, diuretics remain foundational due to their robust blood pressure-lowering capacity and well-established CV outcome benefits. Contemporary reviews and guidelines continue to position diuretics, particularly thiazide and thiazide-like agents, as an essential basis for both monotherapy initiation and combination therapy in hypertension treatment, with benefits that are largely attributable to BP reduction and are supported by long-term CV outcome data [18,26]. Diuretics used in hypertension treatment can be grouped in three different classes: thiazides, loop diuretics, and potassium-sparing agents [27].

Thiazide and thiazide-like diuretics. Compounds of this group (e.g., hydrochlorothiazide, chlorthalidone, indapamide) inhibit Na+ and Cl- reabsorption in the distal convoluted tubule of the renal system and provide reliable BP lowering either as monotherapy or as part of rational combinations. Contemporary analysis emphasizes their long track record of CV risk reduction and practical advantages, while also highlighting clinically relevant pharmacokinetic differences—most notably the longer duration of action of chlorthalidone and indapamide relative to hydrochlorothiazide. The 2023 European Society of Hypertension (ESH) guideline underscores thiazide-like agents as preferred options within the class, reflecting their sustained antihypertensive efficacy and outcome heritage [18,26].

Hypokalemia (abnormally low blood potassium levels) is a common side effect associated with the use of thiazide diuretics; if it develops over the long term, it can lead to cardiac arrhythmia, muscle weakness, polyuria and insulin resistance. In fact, metabolic effects require vigilance. Recent reviews suggest that adverse impacts on glucose homeostasis are most consistently linked to hydrochlorothiazide, supporting the preference for longer-acting thiazide-like compounds when metabolic risk is a concern. Other adverse effects include hypomagnesaemia, hypercalcemia and hyperuricemia [23].

Although it has been reported that thiazides are less effective in patients who consume excessive amounts of salt, have reduced glomerular filtration rate, or take non-steroidal anti-inflammatory drugs, new comparative evidence has clarified some class questions. In a study involving patients with chronic kidney disease and poorly controlled hypertension, treatment with chlorthalidone was found to improve 24 h ambulatory systolic BP control at 12 weeks compared with placebo and to reduce albuminuria, a finding that called into question the traditional view that thiazides are ineffective when glomerular filtration rate is low [28,29].

Loop Diuretics. Commonly used loop diuretics include furosemide, torasemide, azosemide, and bumetanide. They work by reversibly preventing the function of the sodium-potassium-chloride cotransporter in the thick ascending loop of Henle in the kidneys, which is an important site for reabsorption of filtered NaCl. They facilitate a greater exchange of Na^+^ for K^+^ and the subsequent secretion of K^+^. Therefore, loop diuretics elicit antihypertensive effects through excretion-mediated reduction of Na^+^ in extracellular fluid volume, which reduces venous return and decreases cardiac output. Loop diuretics also reduce Ca^2+^ and Mg^2+^ reabsorption in a way that is not yet clear. In addition, loop diuretics promote the production of prostaglandins, which causes renal and venous dilation. Class-typical adverse effects include hypokalemia, hyponatremia, metabolic alkalosis, and hypotension; NSAIDs can attenuate natriuretic/antihypertensive responses via prostaglandin inhibition, underscoring the need to review concomitant therapies [27,30,31].

Potassium-sparing diuretics. Two classes of potassium-sparing diuretics are described: MRAs (spironolactone, eplerenone), which block the mineralocorticoid receptor (MR), thereby preventing the effects of aldosterone, a steroid hormone that increases the reabsorption of Na^+^ and water and the release of K^+^ in the kidney; and epithelial sodium channel (ENaC) blockers (amiloride, triamterene), which also prevent the reabsorption of Na^+^ through these channels. Ultimately, through different mechanisms, both types of compounds reduce distal tubular Na^+^ reabsorption and mitigate kaliuresis (excretion of K^+^ in the urine, often in excessive amounts). MRAs are the guideline-endorsed fourth-line option for resistant hypertension, with robust BP-lowering efficacy but a recognized risk of hyperkalemia, particularly in chronic kidney disease, necessitating careful selection and monitoring. The 2023 ESH guidance recommends spironolactone for resistant hypertension when estimated glomerular filtration rate (eGFR) is ≥30 mL/min/1.73 m^2^ and chlorthalidone as an alternative when eGFR is lower, reflecting both efficacy and safety considerations across kidney function strata [27,32].

Interest in ENaC blockade is growing as either an alternative or adjunct in resistant hypertension. A recent randomized clinical trial compared spironolactone with amiloride and provides contemporary, head-to-head evidence informing the selection of potassium-sparing strategies, complementing established MRA data. Such studies support a tailored approach based on efficacy, tolerability, and hyperkalemia risk [33].

### 3.2. Beta Blockers

β-adrenergic receptor antagonists (β-blockers or BBs) occupy a central position in the modulation of cardiac output, sympathetic drive, and renin release, making them relevant not only for systemic hypertension but also for disorders characterized by heightened neurohormonal activation. β-blockers exert their primary hemodynamic effects by competitively inhibiting β_1_-adrenergic receptors in cardiac tissue, leading to reductions in heart rate, myocardial contractility, and ultimately cardiac output. Their long-term BP–lowering effect is linked to a decrease in peripheral vascular resistance, as well as the suppression of renin release and downstream angiotensin II production, contributing to reduced vasoconstrictor activity. Recent guideline updates from the European Society of Hypertension reaffirm β-blockers as one of the five major antihypertensive drug classes appropriate for initial therapy and combination regimens, while also recognizing their broader utility beyond compelling indications such as heart failure or ischemic heart disease [34,35].

The therapeutic positioning of β-blockers has been supported by contemporary meta-analyses demonstrating that their BP-lowering efficacy is comparable to other first-line agents, including ACEi, ARBs, CCBs, and thiazide-type diuretics. These analyses also confirm significant reductions in CV outcomes in placebo-controlled contexts, although comparisons with other antihypertensive classes show heterogeneous results. Nonetheless, global CV benefit, particularly in patients with sympathetic overactivity or elevated resting heart rate, remains a strong rationale for their use [35,36].

β-Blockers are pharmacologically diverse and are classified according to β_1_-selectivity, lipid solubility, intrinsic sympathomimetic activity, and ancillary α-adrenergic receptor affinity. Non-selective agents may produce undesirable bronchoconstriction or peripheral vasoconstriction due to β_2_-receptor blockade, which is clinically relevant in patients with asthma or peripheral vascular disease. Lipid-soluble β-blockers (e.g., propranolol, metoprolol) more readily cross the blood–brain barrier and have been associated with central nervous system adverse effects, including insomnia, vivid dreams, and mood alterations. This profile highlights the importance of agent selection, especially in individuals susceptible to neuropsychiatric effects. Vasodilating β-blockers (e.g., nebivolol), due to their favorable hemodynamic properties and impact on metabolic parameters, may reduce the incidence of erectile dysfunction, an adverse effect more frequently attributed to non-vasodilating agents [36,37].

Furthermore, real-world clinical practice surveys emphasize contemporary clinician preference for highly β_1_-selective agents such as nebivolol, particularly in regions with high burdens of comorbid hypertension and cardiometabolic disease. These preferences reflect both tolerability advantages and demonstrated effectiveness in lowering BP when used alone or in fixed-dose combinations, especially with ARBs. Such combinations have shown utility in patients with cardiac, renal, respiratory, or metabolic comorbidities, supporting individualized therapeutic strategies [38].

### 3.3. Angiotensin-Converting Enzyme Inhibitors (ACEi)

ACEi play a central role in modulating RAAS-mediated vasoconstriction and neurohormonal activation, mechanisms that are relevant across systemic hypertension and other CV disorders, and constitute a cornerstone of antihypertensive therapy due to their targeted modulation of RAAS. Contemporary international hypertension guidelines consistently position ACEi, alongside ARBs, as first-line therapeutic options for reducing CV morbidity and mortality across diverse patient populations. Their status is supported by robust evidence from randomized controlled trials and meta-analyses demonstrating CV and renal protection that is comparable or superior to other major antihypertensive classes [21,39,40].

ACEi lower BP through inhibition of angiotensin-converting enzyme (ACE), thereby reducing the formation of the potent vasoconstrictor angiotensin II. Additionally, ACE inhibition prevents the degradation of bradykinin, leading to enhanced nitric oxide and prostaglandin release, which further contributes to vasodilation and BP reduction. These pharmacodynamic mechanisms support their efficacy both as monotherapy and as part of combination regimens. Current guidelines, however, discourage combining ACEi with ARBs due to the absence of additional clinical benefit and the risk of adverse renal outcomes when dual RAAS blockade is attempted [21,39,40].

ACEi are generally well tolerated. Common adverse effects include a dry, persistent cough attributable to elevated bradykinin levels, while angioedema remains a rare but clinically significant complication. In susceptible patients, particularly those with renal artery stenosis, ACEi may precipitate acute declines in renal function, underscoring the importance of monitoring renal parameters and potassium levels during therapy. In addition, ACEi have a neutral or favorable metabolic profile. Evidence indicates that ACEi therapy does not adversely affect lipid or glucose metabolism and may even improve insulin sensitivity. This characteristic makes ACEi particularly advantageous in hypertensive patients with metabolic syndrome or a high risk of type 2 diabetes [39,40].

During the COVID-19 pandemic, considerable attention was directed toward the interaction between RAAS inhibitors and ACE2, the cellular receptor facilitating SARS-CoV-2 entry. Early hypotheses suggested potential harm from ACEi/ARB by altering ACE2 expression, prompting speculation regarding therapy modification in infected patients. However, subsequent evidence and guideline-based analyses demonstrated no increased risk and, in some cases, potential benefit associated with continued ACEi/ARB therapy. Observational studies reported trends toward reduced inflammatory markers (such as high-sensitivity C-reactive protein) and lower mortality among ACEi/ARB users, though without consistent statistical significance. As a result, professional societies recommended maintaining ACEi treatment in hypertensive patients with COVID-19 unless specific contraindications emerged [41].

### 3.4. Angiotensin II Receptor Blockers (ARBs)

ARBs provide targeted RAAS modulation through selective AT_1_-receptor antagonism, offering hemodynamic and vascular effects closely aligned with the mechanistic focus of this manuscript. ARBs selectively inhibit the binding of angiotensin II to the angiotensin II type 1 receptor (AT_1_), thereby preventing the downstream effects of RAAS activation.

By antagonizing AT_1_-mediated vasoconstriction, aldosterone secretion, catecholamine release, vasopressin release, and cellular hypertrophy, ARBs effectively reduce systemic vascular resistance and BP. Their receptor-level specificity offers an advantage over ACEi, which exert broader effects through bradykinin modulation, and this selectivity contributes to comparable antihypertensive efficacy with a more favorable tolerability profile. Recent analyses confirm that ARBs demonstrate BP-lowering potency equivalent to ACEi while producing fewer adverse effects such as cough and angioedema [42,43].

ARBs commonly used in clinical practice include candesartan, eprosartan, irbesartan, losartan, olmesartan, telmisartan, and valsartan. They are indicated either as monotherapy or as part of combination therapy with antihypertensive agents other than ACEi. Network meta-analyses comparing these agents have revealed differential but clinically relevant differences across the class: olmesartan and candesartan often rank highest for office and ambulatory BP reduction. In a recent study comparing the efficacy and safety of six ARBs, olmesartan consistently showed the most promising antihypertensive efficacy, and both olmesartan and telmisartan exhibited the lowest probabilities of adverse events. These findings highlight potential therapeutic advantages of certain ARBs in high-risk populations [42].

Beyond BP reduction, ARBs exert beneficial effects on target-organ protection. Evidence demonstrates that ARB therapy contributes to the regression of left ventricular hypertrophy, decreases cerebrovascular risk, and slows the progression of diabetic nephropathy. The class is generally well tolerated; the incidence of cough or angioedema is significantly lower than with ACEi due to the absence of bradykinin accumulation. However, because ARBs modulate the same RAAS pathway, they may still provoke renal function decline in susceptible individuals, particularly those with renal artery stenosis or compromised renal perfusion [43].

### 3.5. Calcium Channel Blockers (CCBs)

CCBs constitute a heterogeneous group of essential agents in the therapeutic management of hypertension, and they modulate vascular tone and myocardial calcium handling, providing antihypertensive efficacy through mechanisms highly relevant to systemic hemodynamic regulation.

Their primary mechanism involves inhibition of voltage-dependent Ca^2+^ channels, predominantly L-type, located in vascular smooth muscle and cardiac myocytes, reducing Ca^2+^ influx and promoting vasodilation with consequent BP lowering. Contemporary classification continues to distinguish two major subclasses: dihydropyridines, characterized by predominant vasoselectivity (e.g., amlodipine, lercanidipine, nicardipine), and non-dihydropyridines, which demonstrate more pronounced cardiac depressant effects (e.g., verapamil, diltiazem). This classical classification is maintained in recent pharmacological and clinical analyses [44].

Dihydropyridine CCBs remain a first-line therapeutic class for use as monotherapy or in combination, given their robust antihypertensive efficacy, metabolic neutrality, and broad tolerability profile [18]. Later-generation dihydropyridines demonstrate improved pharmacokinetic profiles and reduced frequency of adverse effects. Non-dihydropyridines maintain an important role in rate control and the management of supraventricular tachyarrhythmias, reflecting their dual antihypertensive and antiarrhythmic utility. Adverse events are class-specific: dihydropyridines frequently induce peripheral edema, flushing, headache, or reflex tachycardia, whereas non-dihydropyridines may precipitate bradycardia, atrioventricular block, or reduced cardiac output, necessitating cautious use in individuals with conduction abnormalities or heart failure [44,45].

Updated systematic reviews confirm that long-term CCB therapy remains safe and effective, with consistent reductions in stroke risk and acceptable overall tolerability. However, evidence regarding effects on heart failure, myocardial infarction, or transient ischemic attack remains heterogeneous, underscoring the importance of individualized therapy selection [46]. Moreover, guidelines continue to position CCBs as a central pharmacologic option, incorporated into recommended stepwise algorithms and single-pill combinations for the majority of hypertensive phenotypes [18].

### 3.6. Renin Inhibitors

Direct renin inhibition provides a proximal approach to RAAS blockade, targeting the rate-limiting enzymatic step in angiotensin II generation and thereby modulating neurohormonal and hemodynamic pathways central to hypertension.

The clinical effects of ACEi and ARBs underscore the central role of RAAS in BP regulation. Nevertheless, these downstream RAAS inhibitors incompletely suppress compensatory neurohormonal activation, including reactive renin release, thereby limiting full pathway blockade. This pharmacologic limitation stimulated the development of direct renin inhibitors, designed to inhibit the rate-limiting step of RAAS activation by blocking the renin-mediated conversion of angiotensinogen to angiotensin I [47,48].

Aliskiren remains the only orally active, clinically approved direct renin inhibitor for hypertension. It selectively inhibits plasma renin activity, producing dose-dependent reductions in both systolic and diastolic BP, with a tolerability profile comparable to ARBs and placebo in controlled trials. As monotherapy, aliskiren effectively lowers BP; however, multiple studies indicate greater antihypertensive efficacy when combined with other agents such as calcium channel blockers or thiazide diuretics. Despite these hemodynamic benefits, large randomized outcome trials have not demonstrated reductions in major CV or renal endpoints in hypertensive patients treated with aliskiren. Safety concerns, including hyperkalemia and renal impairment, limit its use in combination with ACEi or ARBs, especially in high-risk populations. This finding aligns with contemporary comparative analyses showing that direct renin inhibitors do not outperform ARBs in short-term BP control, adverse events, or mortality outcomes [47,48,49]. The direct renin inhibitor pipeline has narrowed considerably; only imarikiren completed Phase 1 clinical trials, but there is no advanced development for essential hypertension beyond preclinical and early pharmacodynamic studies.

### 3.7. Alpha-Adrenergic Receptor Blockers

α_1_-adrenergic receptor antagonists reduce sympathetically mediated vasoconstriction, contributing to hemodynamic regulation pathways that intersect with other antihypertensive mechanisms described in this review.

α_1_-adrenergic receptors are G-protein-coupled receptors whose subtypes are distributed throughout various human organs and tissues, including the brain, heart, liver, kidneys, spleen, blood vessels, pancreas, and prostate. They mediate a wide range of physiological effects, including neurotransmission, cardiac activity, vasoconstriction, and metabolic regulation. Therefore, they are considered therapeutic targets for the management of various disorders, including hypertension [50].

In fact, selective α_1_-adrenergic receptor antagonists (prazosin, terazosin, and doxazosin) lower BP primarily by inhibiting postsynaptic α_1_-receptors in vascular smooth muscle, thereby reducing sympathetically mediated vasoconstriction and peripheral vascular resistance. This mechanism allows for effective arterial pressure reduction with minimal impact on cardiac output, heart rate, or plasma renin activity. Although historically considered second-line agents, contemporary evidence indicates that α_1_-blockers can be used safely as adjunctive therapy across a wide spectrum of hypertensive patients, particularly in cases of resistant hypertension or when concomitant benign prostatic hyperplasia is present, where these agents provide dual therapeutic benefit. Their use in older adults remains clinically relevant, though systematic reviews highlight increased risks of vasodilation-related adverse events, such as dizziness, postural hypotension, and headache, and emphasize the need for careful monitoring, especially because doxazosin has been associated with a higher incidence of CV events compared with thiazide-based therapy. Consistent with these findings, large meta-analyses continue to caution against the use of α_1_-blockers as monotherapy, citing an elevated risk of acute heart failure despite neutral effects on overall CV and all-cause mortality. Nevertheless, when used judiciously as add-on therapy, particularly in patients with coexisting lower urinary tract symptoms, selective α_1_-antagonists remain a clinically valuable component of individualized hypertension management [51,52,53].

### 3.8. Direct Vasodilators

Direct vasodilators act through potent arteriolar smooth-muscle relaxation, contributing to blood pressure reduction via mechanisms that interact with sympathetic activation, renal sodium handling, and vascular remodeling. The main representatives of this pharmacological class, hydralazine and minoxidil, remain potent antihypertensive agents that exert their effects through direct relaxation of arteriolar smooth muscle.

Minoxidil acts as an ATP-sensitive potassium channel opener, producing marked arteriolar dilatation and substantial reductions in peripheral vascular resistance. Hydralazine induces arterial vasodilation through inhibition of intracellular calcium-dependent contractile mechanisms, contributing to its established role in refractory hypertension and specific populations such as patients with heart failure with reduced ejection fraction (HFrEF). Neither minoxidil nor hydralazine produces significant venous dilation, and both agents evoke compensatory neurohumoral responses such as activation of the sympathetic nervous system and renin–angiotensin system, which contribute to reflex tachycardia, increased cardiac output, and sodium and water retention. These counterregulatory mechanisms often necessitate concomitant therapy with β-blockers and diuretics to achieve adequate BP control. In contemporary practice, direct vasodilators are generally reserved for severe or resistant hypertension, reflecting their potent antihypertensive efficacy but also their unfavorable adverse-effect profiles, including fluid retention, reflex tachycardia, and, in the case of hydralazine, risks such as drug-induced lupus erythematosus [54,55].

Recent experimental research has raised additional concerns regarding long-term use of direct vasodilators, demonstrating that both minoxidil and hydralazine may aggravate abdominal aortic aneurysm progression through enhanced renin–angiotensin activation and vascular inflammatory responses—findings that emphasize the importance of careful patient selection and ongoing monitoring when employing these agents. Despite these limitations, hydralazine and minoxidil retain a defined therapeutic niche as third-line or adjunctive therapies when multiple standard antihypertensive classes fail to achieve BP control [56].

### 3.9. Endothelin Receptor Antagonists (ERAs)

ERAs target one of the most powerful vasoconstrictor pathways involved in resistant hypertension, with mechanisms that overlap key vascular processes relevant to systemic and pulmonary vascular disease.

The endothelin system plays a pivotal role in increasing peripheral vascular resistance, the final pathway in the pathogenesis of hypertension. Endothelin-1 (ET-1), a 21-amino acid peptide produced by vascular endothelial cells, is the major active endothelin isoform and a key contributor to increased vascular tone and hypertension. ET-1 acts predominantly through ETA and ETB, two G-protein–coupled transmembrane receptors widely distributed throughout the CV system, promoting potent and sustained vasoconstriction as well as vascular remodeling, thereby contributing to resistant hypertension. ET-1 overexpression drives adverse vascular remodeling. Patients with hypertension exhibit heightened ET-mediated vascular activity, leading to pronounced vasoconstriction that can be reversed through non-selective blockade of both ETA and ETB receptors. In addition to promoting vasodilation, this dual blockade may exert anti-inflammatory and antifibrotic effects and contribute to the reversal of cardiac hypertrophy [8,57].

Thus, ERAs represent a novel therapeutic class targeting the ET-1 pathway. Recent clinical trials have demonstrated that dual ERAs, particularly the newly approved agent aprocitentan, effectively reduce BP in patients with resistant hypertension, with sustained effects and good tolerability across diverse subgroups, including older adults, individuals with diabetes, and those with chronic kidney disease [58]. Moreover, pivotal evidence shows that aprocitentan produces clinically meaningful and durable reductions in BP compared with placebo, reinforcing its role as an emerging option when conventional therapies fail [59,60].

Additionally, ERAs hold potential in secondary hypertension associated with targeted cancer therapies, where excessive activation of the endothelin axis contributes to tyrosine kinase inhibitor-induced hypertension. Collectively, contemporary data position endothelin receptor antagonism as a promising strategy for refractory and mechanism-specific hypertension, expanding the therapeutic landscape beyond traditional antihypertensive classes. In general, ERAs are associated with several adverse effects, including headache, flushing, edema, and anemia. These agents are also classified as pregnancy category X, as studies conducted in knockout mouse models have demonstrated their teratogenic potential [57,61,62].

**Table 1:** Summary of more used antihypertensive drugs. Information mainly compiled from the summary product characteristics of the drugs and from the references cited.

Class	Drugs	Mechanism	Adverse Effects	Refs.
**Diuretics**				[21,27]
Thiazide diuretics	bendroflumethiazide, chlorothiazide, chlorthalidone, hydrochlorothiazide, indapamide, polythiazide, trichlormethiazide	Inhibit the reabsorption of Na^+^/Cl^−^ in the early distal convoluted tubule of the nephrons	Hypokalemia Hypomagnesemia Hypercalcemia Hyperuricemia	
Loop diuretics	azosemide, bumetanide, furosemide, torasemide,	Reversible inhibition of Na^+^/K^+^/Cl^−^ co-transporter in the ascending loop of Henle of nephron	Hyponatremia Hypokalemia Metabolic alkalosis, Hypovolemia Hypotension	
K-sparing diuretics	amiloride, triamterene	Epithelial Na channel blockers Decrease Na reabsorption	Hyperkalemia	
	spironolactone, eplerenone	Mineralocorticoid receptor antagonists Aldosterone receptor blockade Decrease Na reabsorption	Hyperkalemia Gynecomastia (spironolactone)	
**β-blockers**				[21,34,35]
Non-vasodilating & β_1_-selective	acebutolol, atenolol, betaxolol, bisoprolol, esmolol, metoprolol	Blockade of cardiac β_1_ receptors reduce heart rate and cardiac contractility. Also inhibit the release of renin	Bradycardia Hypotension Reduced left ventricular contractility Bronchospasm. Impair lipid, glucose and insulin metabolism Depression Lethargy Vivid dreams Constipation Impotence	
Non-vasodilating & no β_1_ selective	carteolol, nadolol, oxprenolol, penbutolol, propranolol, sotalol, timolol, pindolol			
Vasodilating & β_1_-selective	nebivolol,	Vasodilation mediated via β_3_ agonism and NO release		
Vasodilating & no β_1_-selective	carvedilol	Vasodilation mediated via α_1_ receptor antagonism		
**Angiotensin-converting** **enzyme** **inhibitors**	benazepril, captopril, cilazapril, enalapril, fosinopril, imidapril, lisinopril, moexipril, perindopril, quinapril, ramipril, trandolapril, zofenopril	Block conversion of angiotensin I to angiotensin II (a potent vasoconstrictor) and subsequently the production of aldosterone	Paroxysmal cough Angioedema Renal failure Hyperkalemia	[21,40]
**Angiotensin II receptor** **blockers**	candesartan, eprosartan, irbesartan, losartan, olmesartan, telmisartan, valsartan	Competitive antagonism of angiotensin II receptor 1 avoiding the vasoconstrictor effect of angiotensin II	Paroxysmal cough Angioedema Hypotension Hyperkalemia	[21,42]
Ca^2+^ channel blockers				[21,44]
Dihydropyridines	amlodipine, clevidipine, felodipine, isradipine, lacidipine, lercanidipine, manidipine, nicardipine, nifedipine, nitrendipine	Reduced flow of calcium to vascular smooth muscle, reducing contraction efficiency and relaxing the vasculature	Peripheral edema Headache Flushing Tachycardia Constipation Bradycardia	
Non-dihydropyridines	diltiazem, verapamil			
**Direct renin** **inhibitors**	aliskiren	Inhibits the conversion of angiotensinogen to angiotensin, via renin inhibition	Hyperkalemia Renal impairment Fatigue Headache Dizziness Diarrhea Nasopharyngitis Back pain	[48,49]
**α-adrenergic** **receptor** **blockers**	doxazosin, prazosin, terazosin	Block the α1 adrenoreceptors on vascular smooth muscles	Dizziness Headache Nausea & drowsiness. Concerns of worsening heart failure	[21,51]
**Direct** **vasodilators**	hydralazine, minoxidil	Relaxation of vascular smooth muscle, primarily arterioles	Reflex tachycardia Fluid retention. Nausea & vomiting Headache Joint and chest pain	[21,63]
**Endothelin (ET) receptor** **antagonists**	aprocitentan	Dual antagonism in ET receptors ETA & ETB, avoiding vasoconstriction effects of ET	Edema, fluid retention, ↓ Hemoglobin ↑ Aminotransferases, ↓ Sperm counts Embryo-fetal toxicity,	[58,62]

↑ means an increase in levels and ↓ means a decrease.

### 3.10. New Targets for Hypertension Treatment

The search for novel antihypertensive targets reflects the need to overcome limitations of current therapies and address persistent neurohormonal activation, renal hemodynamic dysregulation, sympathetic overactivity, and vascular remodeling in resistant hypertension. Despite the number of drugs available, effective management of hypertension, one of the most prevalent CVDs worldwide, remains difficult in the resistant hypertensive population. Considering, in addition, that antihypertensive drugs can have adverse effects, it is still necessary to search for new therapeutic targets and treatments to control hypertension and its comorbidities. In the past, classic drug targets such as the aldosterone receptor, aldosterone synthase, as well as other receptors and mediators involved in RAS regulation have been investigated. Recently, vaccines and drugs targeting the gastrointestinal microbiome, representing *classes* of drugs, have also been investigated for the management of BP. The main or most studied potential targets and their drugs are listed in Table 2.

New agents currently at a relatively advanced stage of clinical development and which have shown strong evidence of efficacy in the treatment of high BP can be summarized into three main groups: aldosterone inhibitors, new biotechnological agents that act on the renin-angiotensin system, and aminopeptidase inhibitors.

New Ca^2+^ channel blockers. However, regarding the currently used Ca^2+^ channel blockers, beyond the L-type channel, emerging evidence has underscored the physiological and therapeutic relevance of T-type and N-type Ca^2+^ channels, which regulate renin and aldosterone secretion (T-type) and sympathetic neurotransmitter release (N-type). Compounds with dual L/N or L/T inhibitory activity, such as cilnidipine (L/N) and benidipine, manidipine, efonidipine, or azelnidipine (L/T), exhibit additional organ-protective effects, including attenuation of sympathetic overactivity, improvement of renal hemodynamics, and reduction of proteinuria, particularly when combined with renin–angiotensin system blockers. Clinical reviews highlight cilnidipine’s favorable effects on renal blood flow, reduced reflex tachycardia, and improved tolerability relative to traditional L-type agents. Although largely marketed in Asia-Pacific regions, these agents represent a promising therapeutic avenue for hypertensive patients with chronic kidney disease, although long-term comparative trials remain ongoing to confirm sustained renoprotection [45,64].

Aldosterone. Among its various biological actions, aldosterone plays a central role in the regulation of Na^+^ excretion, fluid volume homeostasis, and vascular tone. Its effects are mediated through activation of the mineralocorticoid receptor (MR); therefore, MR blockade—using antagonists such as spironolactone or eplerenone, as discussed in previous sections—abolishes the detrimental consequences of aldosterone and improves CV outcomes, including BP reduction in patients with resistant hypertension. However, the long-term use of MR antagonists is limited by their suboptimal tolerability profile. This limitation has stimulated the development of new selective, non-steroidal MR antagonists, including finerenone (marketed in Europe and the USA), esaxerenone (marketed in Japan), ocedurenone, and balcinrenone (in development), as alternatives to spironolactone [65]. Furthermore, MR blockade may elicit a counterregulatory response by inducing an increase in plasma renin and aldosterone concentrations, thereby partially offsetting treatment benefits.

To mitigate these effects, a therapeutic strategy targeting the inhibition of aldosterone synthesis has emerged, specifically through suppression of a key enzyme in this pathway, aldosterone synthase (CYP11B2). Recent meta-analyses of studies evaluating new aldosterone-synthesis inhibitors demonstrate clinically meaningful reductions in BP, albeit accompanied by an increased risk of hyperkalemia. The most advanced compounds (baxdrostat, lorundrostat, and dexfadrostat) exhibit improved selectivity over CYP11B1 [66,67,68].

Biologic therapies of RAAS modulation. In recent years, a new generation of biologically derived antihypertensive therapies has been developed by modulating proximal components of the RAAS through RNA interference (RNAi) or related technologies. These strategies aim to overcome the limitations of conventional antihypertensive agents, such as suboptimal adherence and fluctuations in BP control. Within this group, zilebesiran, a small interfering RNA (siRNA) directed against hepatic angiotensinogen, is the most advanced agent and represents a paradigm shift in the management of hypertension [69]. By degrading angiotensinogen mRNA, zilebesiran markedly and sustainably reduces the production of this peptide, which is the initial substrate of the RAAS. This leads to a profound and durable reduction in circulating angiotensin II levels and to a sustained lowering of BP for up to 24 weeks following a single subcutaneous injection [70].

The phase I–II clinical development of zilebesiran, particularly the KARDIA-1 and KARDIA-2 studies, has demonstrated >90% suppression of angiotensinogen, significant reductions in systolic BP, sustained effects lasting up to 6 months per dose, and good tolerability with few clinically relevant adverse events [71,72]. In the phase III KARDIA-3 trial, according to the developer’s announcement, which evaluated patients with uncontrolled hypertension and high CV risk, a significant reduction in systolic BP at 3 months with the 300 mg dose was achieved, with benefits sustained up to 6 months and an encouraging safety profile, even when combined with multiple antihypertensive agents [73].

The available data suggest that zilebesiran could transform the management of hypertension by improving adherence through a regimen of two injections per year, achieving stable and sustained BP control thanks to prolonged angiotensinogen suppression, which ensures continuous RAAS modulation and prevents fluctuations. Moreover, although these findings are preliminary, emerging evidence points to potential additional benefits in the heart, kidneys, and retina through reduction of hemodynamic load and RAAS-mediated stress.

Aminopeptidase. Aminopeptidase A (APA) inhibitors represent an emerging therapeutic approach aimed at modulating the activity of the RAS at the central level. APA catalyzes the conversion of angiotensin II into angiotensin III, and this peptide exerts a significant pressor effect within the brain RAS. APA inhibition reduces the formation of angiotensin III and attenuates central sympathetic and vasopressor activity, leading to a decrease in BP [74,75].

EC33 is a potent inhibitor of brain APA that, in animal models, resulted in a reduction in BP. To facilitate the passage of EC33 across the blood–brain barrier, two EC33 molecules were linked via a disulfide bridge to form a prodrug known as firibastat. In preclinical studies, firibastat demonstrated significant reductions in BP, supporting its potential as a centrally acting antihypertensive therapy [76,77]. In clinical research, phase I–II studies of firibastat showed an acceptable safety profile and moderate reductions in BP among patients with hypertension, which led to its evaluation in resistant hypertension. However, results from the phase III FRESH trial did not demonstrate significant reductions in BP compared with placebo, calling into question its utility in patients with difficult-to-control hypertension [78,79].

Overall, although the pathophysiological rationale supports APA inhibitors as a novel and biologically sound strategy to modulate the central RAS, the available clinical evidence indicates limited efficacy in resistant hypertension. Their safety profile is considered acceptable, but the lack of consistent clinical benefits has prevented their incorporation into current therapeutic practice.

**Table 2:** Some of the key drugs in clinical development for the treatment of high blood pressure.

Target	Drug	Mode of Action	Status	Key Safety Signs	Refs.
New Ca^2+^ channel blockers	aramidipine, azelnidipine, benidipine, cilnidipine, efonidipine, manidipine, nilvadipine	Dual blockade of T/N/L-type Ca^2+^ channels	Approved and marketed mainly in Asia-Pacific area	Peripheral edema Headache Flushing (class effect)	[64,80]
Aldosterone	baxdrostat lorundrostat; dexfadrostat	Blockade CYP11B2 enzyme Aldosterone synthesis inhibition Reduction of Na retention and volume expansion	Phase II/III trials showing consistent reductions in blood pressure.	Hyperkalemia Monitoring of K levels and renal function Cortisol insufficiency Hypotension Hyponatremia.	[66,67,68]
Renin–angiotensin system	zilebesiran	*si*RNA targeting hepatic angiotensinogen	Phase II/III Quarterly/half-yearly Dosing in evaluation	Good safety profile in early stages; long-term follow-up ongoing	[69,70]
Amino peptidase	firibastat	Brain aminopeptidase inhibition. Reduction of angiotensin III production	Phase III	Headaches and reversible skin reactions.	[75,78]

### 3.11. Antihypertensive Agents and Pulmonary Vascular Disease

Antihypertensive pharmacotherapy is grounded in the modulation of key biological pathways that extend beyond systemic blood pressure control and are equally relevant to pulmonary vascular pathophysiology. Central mechanisms such as RAAS activation, endothelin signaling, NO–sGC dysregulation, sympathetic overactivity, and calcium-dependent vascular tone are shared across systemic and pulmonary circulations, where they contribute to vasoconstriction, vascular remodeling, and right ventricular overload. Accordingly, several therapeutic classes reviewed in this section, including RAAS inhibitors, endothelin receptor antagonists, and sGC pathway modulators, have direct or potential applicability in pulmonary vascular disease, either through established indications or through ongoing translational research.

## 4. Antiarrhythmics

Antiarrhythmic drugs (AADs) remain an essential component of modern arrhythmia management despite the rapid evolution of catheter-based and device-based therapies. Their clinical use is fundamentally constrained by a narrow therapeutic window, i.e., the dose range in which a drug can be used without causing adverse effects. Therefore, small deviations from the optimal dose can lead to significant toxicity. This narrow margin results largely from the complex electrophysiological substrates that AADs target, as well as from inter-individual variability in drug metabolism, ion-channel expression, and structural heart disease. Recent reviews highlight that AADs may exert adverse cardiac and extracardiac effects that are often unrelated to their primary electrophysiologic targets, further complicating therapeutic decision-making [81,82].

Extracardiac toxicities may include anticholinergic manifestations (e.g., constipation, xerostomia, urinary retention), thyroid and hepatic dysfunction with certain multichannel blockers such as amiodarone, and neurological or gastrointestinal effects depending on the drug class. Of particular clinical concern is proarrhythmia, an iatrogenic phenomenon in which treatment intended to suppress arrhythmias paradoxically precipitates new or more malignant ventricular or atrial arrhythmias. Notably, proarrhythmia can develop not only at supratherapeutic concentrations but also at standard therapeutic levels, reflecting the highly sensitive nature of cardiac ion-channel dynamics and the heterogeneity of diseased myocardium [81,82].

Although catheter ablation has revolutionized the treatment of many arrhythmias, particularly atrioventricular nodal reentrant tachycardia, accessory pathway–mediated tachycardias, and selected forms of atrial flutter, large patient groups still rely on pharmacologic therapy. The management of atrial fibrillation (AF) and ventricular tachycardia (VT) arising in structurally abnormal hearts remains especially challenging. Ablation strategies for persistent AF and scar-related VT do not guarantee long-term freedom from recurrence, and procedure-related risks or comorbidities may render some patients unsuitable candidates for interventional management. For this reason, the European Heart Rhythm Association (EHRA) emphasizes that AADs continue to play a critical role as primary therapy, as adjuncts to ablation, and as back-up options when procedural approaches are not feasible or are only partially effective [83].

The contemporary therapeutic landscape therefore requires a nuanced, individualized risk-benefit assessment. Clinicians must consider not only the electrophysiologic profile of each agent but also comorbidities, potential drug–drug interactions, QRS and QT interval responses, renal and hepatic function, and long-term toxicities. Modern classification systems, updated to reflect advances in molecular electrophysiology, recognize that many AAD exert multichannel effects and cannot be strictly categorized within a traditional schema. Nonetheless, for practical clinical use, AADs continue to be grouped by their predominant mechanisms of action, which are summarized in Table 3.

### 4.1. Class 0. Pacemaker Channel Blockers

The newly defined Class 0 encompasses a single subclass of agents that act on hyperpolarization-activated, cyclic nucleotide-gated (HCN) channels, inhibiting the I_f_ current, a critical determinant of sinoatrial nodal pacemaker activity, particularly during the early phase of diastolic depolarization, and of pacemaker function in both the sinoatrial and atrioventricular nodes. HCN channels are also expressed in ventricular Purkinje fibers and, potentially, in other cardiac cell populations capable of spontaneous depolarization. Ivabradine, the sole agent currently within this subclass, is employed to reduce inappropriate sinus tachycardia or when sinus tachycardia accompanies heart failure [84].

### 4.2. Class I. Sodium Channel Blockers

Class I antiarrhythmic agents are rapid sodium (Na^+^) channel blockers that decrease the slope of phase 0 of the myocardial action potential, thereby reducing conduction velocity. They are subdivided according to their channel binding and unbinding kinetics into Class IA, IB, and IC, corresponding to intermediate, rapid, and slow blockade, respectively [85,86,87].

Class IA: quinidine, procainamide, disopyramide. These agents block Na^+^ channels and also inhibit K^+^ channels, resulting in prolonged repolarization and QT-interval prolongation, with an associated risk of *torsade de pointes*. They may reduce contractility and worsen hearth failure (HF). Procainamide is used in supraventricular arrhythmias, particularly in reentrant tachycardias, although its clinical use has declined because of adverse effects and reduced availability.

Class IB: lidocaine and mexiletine. These drugs have a higher affinity for inactivated Na^+^ channels, making them more effective in ischemic tissue and in ventricular tachyarrhythmias. Lidocaine is useful in ventricular arrhythmias associated with ischemia or acute myocardial infarction. Mexiletine, an oral analogue, is used as an adjunct in ventricular arrhythmias and in channelopathies such as LQT3, although its use as monotherapy is uncommon. Notable adverse effects include neurological symptoms (confusion, seizures) and gastrointestinal intolerance.

Class IC: flecainide and propafenone. These are potent Na^+^ channel blockers with slow dissociation kinetics, leading to marked QRS prolongation. They exert minimal effects on repolarization and do not prolong the QT interval. They exhibit pronounced use-dependence, with their electrophysiologic effects increasing at higher heart rates. These agents may induce ventricular tachycardia and are contraindicated in structural heart disease, particularly in coronary artery disease or reduced left ventricular ejection fraction.

### 4.3. Class II. Beta Blockers

As has already been commented in various sections of this report, β-blockers (BBs) have multiple effects on cardiac electrical activity. They exert multiple electrophysiological effects on cardiac tissue, primarily through competitive antagonism of β-adrenergic receptors, thereby attenuating sympathetic stimulation of the myocardium. This mechanism underlies their well-established antiarrhythmic properties, including suppression of enhanced automaticity, reduction of triggered activity, and stabilization of conduction in adrenergically driven arrhythmias. Importantly, in contrast to Class I and Class III antiarrhythmic drugs, β-blockers have a favorable proarrhythmic profile, with a very low propensity to induce malignant ventricular arrhythmias [88].

Clinically, BBs play a pivotal role in reducing mortality after myocardial infarction and in improving outcomes in heart failure, where sympathetic overactivation contributes to arrhythmogenesis and disease progression. Even in the contemporary era of implantable cardioverter-defibrillators, BBs remain first-line therapy both for the management of underlying structural heart disease and for the prevention of recurrent arrhythmias that may otherwise require device intervention. Their utility extends to atrial fibrillation, where they are widely used for ventricular rate control and for lowering the risk of arrhythmia recurrence [89,90].

BBs constitute a heterogeneous pharmacological class, differing significantly in β_1_-selectivity, intrinsic sympathomimetic activity, lipid solubility, elimination half-life, and ancillary properties such as vasodilation. These pharmacokinetic and pharmacodynamic distinctions are clinically meaningful and guide the selection of the most appropriate agent for each therapeutic scenario. Far from being secondary options, β-blockers provide a robust efficacy-to-safety balance, making them essential components of therapy across a wide spectrum of cardiac arrhythmias.

### 4.4. Class III. Potassium Channel Blockers

Class III antiarrhythmic agents, including amiodarone, dronedarone, sotalol, ibutilide, and dofetilide, exert their primary electrophysiologic effect by blocking repolarizing potassium currents, thereby prolonging action-potential duration and refractoriness without significantly affecting intracardiac conduction velocity [91].

Amiodarone exhibits multichannel blockade and possesses additional class I and II properties, including inhibition of inactivated sodium channels at higher heart rates and non-competitive antagonism of β-adrenergic receptors. It remains the most effective AAD for both atrial and ventricular arrhythmias, with a relatively low incidence of torsades de pointes despite marked QT prolongation. Nonetheless, its use is limited by a broad array of dose-dependent systemic toxicities, which may involve the thyroid gland, lungs, liver, skin, eyes, gastrointestinal tract, and nervous system. Pulmonary toxicity, including interstitial pneumonitis, organizing pneumonia, acute respiratory distress syndrome, and pulmonary fibrosis, is one of the most clinically significant and potentially fatal complications, often presenting insidiously and requiring prompt recognition and drug discontinuation. Because of these severe adverse effects, amiodarone is typically reserved for clinically significant ventricular arrhythmias or when other AADs are ineffective or contraindicated [92,93,94].

Dronedarone, a non-iodinated structural analogue of amiodarone, was developed to reduce thyroid and pulmonary toxicities. Like amiodarone, it demonstrates multichannel activity, including class II and IV effects. Contemporary guidelines recommend its use for maintaining sinus rhythm in patients with paroxysmal or persistent atrial fibrillation, but contraindicate it in those with symptomatic heart failure, permanent atrial fibrillation, or recent decompensation due to increased mortality risk. Compared with other class III agents, dronedarone and amiodarone are associated with a lower incidence of polymorphic ventricular tachycardia, attributed to their more homogeneous electrophysiologic effects across myocardial tissue [95].

Sotalol exhibits both non-selective β-adrenergic blockade (class II) and potassium channel blockade (class III). Its reverse-use dependence leads to more pronounced action-potential prolongation at slower heart rates, thereby increasing the risk of torsades de pointes, particularly in patients with bradycardia or concomitant QT-prolonging medications. Recent clinical investigations confirm that although outpatient initiation can be safe under structured ECG monitoring, sotalol continues to pose a quantifiable risk of QT prolongation requiring vigilant surveillance [96,97].

Ibutilide and dofetilide are “pure” class III antiarrhythmic agents that selectively block the rapid component of the delayed rectifier potassium current (IKr). Their reverse-use dependence and potent QT-prolonging effects predispose to torsades de pointes, necessitating strict inpatient monitoring during initiation and dose titration—particularly in patients with renal impairment or baseline QT prolongation. Both agents remain effective options for pharmacologic cardioversion of atrial fibrillation or flutter, though clinical use is limited by these proarrhythmic risks [98,99].

### 4.5. Class IV. Calcium Channel Blockers

Non-dihydropyridine calcium channel blockers, specifically verapamil and diltiazem, exert their antiarrhythmic activity by selectively inhibiting L-type Ca^2+^ channels within the sinoatrial and atrioventricular nodes, thereby increasing AV-nodal refractoriness, slowing conduction, and reducing nodal automaticity. These electrophysiologic actions make them effective agents for ventricular rate control in atrial fibrillation and for the termination or prevention of AV node-dependent supraventricular tachycardias. Because their negative inotropic effects can precipitate hemodynamic deterioration, these types of Ca^2+^ channel blockers are contraindicated in patients with heart failure with HFrEF, where guideline-based recommendations advise avoidance due to risk of clinical worsening [100,101].

### 4.6. Class V

Digoxin, a cardiac glycoside, exerts its pharmacologic effect through reversible inhibition of the Na^+^/K^+^-ATPase, leading to increased intracellular sodium, reduced Na^+^/Ca^2+^ exchange, and subsequent augmentation of intracellular calcium, thereby enhancing positive inotropy. It also increases vagal tone, resulting in suppression of sinoatrial node automaticity and prolongation of atrioventricular nodal refractoriness, which contributes to its utility in ventricular rate control in atrial fibrillation. Digoxin possesses a narrow therapeutic index, and toxicity is particularly common in the setting of renal impairment, electrolyte derangements, or drug–drug interactions. Clinical manifestations range from gastrointestinal symptoms (nausea, vomiting, diarrhea) and visual disturbances to life-threatening arrhythmias, including atrial tachycardia with block, bidirectional ventricular tachycardia, and high-grade AV block. Although its use has declined in adults in favor of β-blockers and non-dihydropyridine calcium-channel blockers, digoxin continues to play a role in selected patients, including pregnant women and pediatric populations, provided careful monitoring is ensured [102,103].

Adenosine acts by activating A1-adenosine receptors in the atrioventricular node, which opens inward-rectifier potassium channels and inhibits calcium influx, resulting in profound but transient AV nodal block. It may also shorten atrial refractory periods, occasionally precipitating atrial fibrillation or generating transient ventricular ectopy immediately following administration. Adenosine is considered the first-line pharmacologic therapy for hemodynamically stable paroxysmal supraventricular tachycardia (PSVT), including AV nodal reentrant tachycardia and AV reentrant tachycardia. Despite its utility, adenosine may provoke severe bronchospasm in patients with active obstructive airway disease and is absolutely contraindicated in pre-excited atrial fibrillation (Wolff–Parkinson–White syndrome) due to the risk of accelerating conduction over the accessory pathway and precipitating ventricular fibrillation. Its brief duration of action and diagnostic value make adenosine particularly advantageous in both adult and pediatric paroxysmal supraventricular tachycardia, with contemporary evidence confirming its excellent safety profile in children [104,105,106].

**Table 3:** Summary of antiarrhythmic drugs. Table compiled from information in [84,107,108].

Class	Known as	Examples	Mechanism	Clinical Uses	Main Noncardiac Adverse Effects
0	Pacemaker channel blockers	Ivabradine	HCN channel modulators, blocking I_f_ current, in sinoatrial node	Inappropriate sinus tachycardia Rate control in HF	Bradycardia Hypertension
Ia	Na^+^ channel blockers	Quinidine Procainamide Disopyramide	Na^+^ channel blockade K^+^ channel blocking effect Affect QRS complex Prolong the AP Intermediate effect depolarization initiation	Ventricular dysrhythmias Prevention of recurrent paroxysmal AF	Diarrhea (quinidine) Prostatism Glaucoma (disopyramide) Arthritis (chronic procainamide)
Ib		Lidocaine Mexiletine	Na^+^ channel blockade Overdose prolongs QRS complex Shorten the AP Weak effect on depolarization initiation	Treatment and prevention of VT and fibrillation during and immediately after myocardial infarction	Tremor (mexiletine)
Ic		Flecainide Propafenone	Na^+^ channel blockade No effect on AP Strongest effect on the depolarization initiation	Prevent paroxysmal AF (flecainide) Recurrent tachyarrhythmias	Asthma Peripheral vascular disease Hypoglycemia (propafenone)
II	Beta blockers	See Table 1	β-Adrenoceptor antagonism Propanolol shows some Na^+^ channel-blocking effects	Reduce mortality following myocardial infarction Rate control in AF Prevent recurrence of tachyarrhythmias provoked by increased sympathetic activity. VT prevention	See Table 1
III	K^+^ channel blockers	Amiodarone Sotalol Ibutilide Dofetilide Dronedaron	K^+^ channel blockade Prolongation of repolarization Multichannel effects Sotalol also a β blocker Amiodarone has Class III mostly, but also, I, II, & IV activity	AF maintenance VT management WPWS. VT and AF (sotalol). Atrial flutter and AF (ibutilide) Prevent paroxysmal AF & hemodynamically stable VT (amiodarone)	Thyroid/liver issues Bradycardia Lung disease (amiodarone)
**IV**	Ca^2+^ channel blockers	Verapamil Diltiazem See table	L-type Ca^2+^ channel blockade	Prevent recurrence of paroxysmal SVT Rate control in AF Reduce the ventricular rate in patients with AF, provided they do not have WPWS or a related disorder.	Constipation (verapamil) Edema
**V**	Other	Adenosine Digoxin Magnesium sulfate	Work by other or unknown mechanisms: direct nodal inhibition, membrane stabilization	SVT termination Slow ventricular rate in rapid persistent AF AF rate control in HF patients who remain symptomatic despite optimal use of diuretics and ACEi	Nausea, vomiting & diarrhea, Changes in color vision (digoxin) Persistent bronchoconstriction (adenosine)

AF: Atrial fibrillation; AP: action potential; HCN: hyperpolarization-activated cyclic nucleotide gated; HF: heart failure; ACEi: angiotensin-converting enzyme inhibitors; QRS complex: the combination of three of the graphical deflections seen on a typical electrocardiogram; SVT: supraventricular tachycardia; VT: ventricular tachycardia; WPWS: Wolff–Parkinson–White syndrome.

### 4.7. Potential Drugs, Candidates and Targets

After several years with limited pharmacological innovation in the field of arrhythmia, a new group of compounds in development has recently emerged, reaching cinical phase II and III. These agents include molecules with novel mechanisms of action targeting previously unexplored ion channels, as well as new pharmaceutical formulations designed to facilitate treatment and improve medication adherence. A summary of these compounds is presented in Table 4.

Etripamil, an ultrarapid-acting L-type calcium channel blocker, is being developed as an intranasal spray intended for self-administration during episodes of supraventricular tachycardia (SVT). Clinical trials have demonstrated significantly higher conversion rates compared with placebo, with a favorable safety profile and predominantly local effects. Additionally, it has shown utility for rapid ventricular rate reduction in AF [109]. Regarding new delivery systems, an inhaled formulation of flecainide, a drug currently administered orally, is also under development. This formulation achieves much faster conversion of recent-onset AF; however, further optimization is required to improve bioavailability.

Among molecules targeting novel pathways, AP30663 stands out as an inhibitor of small-conductance Ca^2+^-activated K^+^ channels [K(Ca)2]. These channels are expressed throughout the organism and contribute to action potential repolarization. Numerous studies in both small and large animal models have provided robust evidence supporting the antiarrhythmic properties of K(Ca)2 channel blockers. Moreover, their expression and function may be altered in various CV pathological contexts. Early studies indicate effectiveness in AF conversion and acceptable safety, although QT prolongation necessitates the development of second-generation molecules with greater selectivity [82].

In the area of highly specific targets, two-pore domain K^+^ (K_2P_) channels generate an instantaneous, non-inactivating leak current (I_K2P_) that stabilizes the membrane potential and regulates cardiomyocyte excitability. Blockade of the K_2P_ channels predominantly expressed in atrial tissue (TWIK-1, TASK-1 and TREK-1) prolongs atrial action potential duration, which could destabilize arrhythmias. In patients with AF and in animal models of AF, TASK-1 expression has been consistently reported to be increased [82]. Doxapram, a TASK-1 channel inhibitor, is currently under investigation for AF cardioversion.

Another emerging approach involves new multichannel-blocking agents, as the complexity of arrhythmogenic mechanisms means single-channel targeting is often insufficient for achieving optimal therapeutic efficacy. Sulcardine (HBI-3000) is an agent that acts on multiple currents (I_Na_, I_CaL_, I_Kr_), exhibiting a broad antiarrhythmic profile and a low likelihood of *causing torsades de pointes*. Initial studies demonstrated its potential for converting AF resistant to other agents [110,111].

Additionally, certain drugs with less innovative mechanisms but distinct therapeutic strategies remain under evaluation. Bucindolol, a β-blocker with α-blocking properties, has shown efficacy modulated by specific genetic polymorphisms (ADRB1 Arg389Arg), opening the door to genotype-guided therapy [112]. Budiodarone, an amiodarone analogue with a shorter half-life, has demonstrated reduced AF recurrence in preliminary studies [113].

AF has been shown to induce remodeling and loss of contractile function, at least in part through activation of histone deacetylase 6 (HDAC6), an enzyme regulating acetylation of both histones and cytosolic proteins, leading to disrupted α-tubulin proteostasis and microtubule disorganization within cardiomyocytes. In vivo HDAC6 inhibition protects against AF-related atrial remodeling, highlighting HDAC6 as a potential therapeutic target in clinical AF. HDAC6 inhibitors, such as PKN605, represent an entirely new therapeutic class, linked not only to ion-channel modulation but also to atrial structural and electrical remodeling. Preclinical studies and early human trials confirm their biological activity and potential as disease-modifying therapy [114].

**Table 4:** Candidates in clinical stages of development, with potential indications in tachycardias and arrhythmias (new formulations under development, such as inhaled flecainide, are not included).

Drug Name	Indication	Clinical Phase	Mechanism of Action	Ref.
Etripamil	SVT AF	III	L-Type Ca^2+^ Channel Blocker	[109]
AP30663	AF	II	Small-conductance Ca^2+^-activated K^+^ channel (KCa2) inhibitor	[115]
AP31969	sinus rhythm maintenance (AF)	I	Small-conductance Ca^2+^-activated K^+^ channel (KCa2) inhibitor	[116]
Sulcardine (HBI-300)	VT & AF	I	Multiple ion channel blocker (*I*_Na,P_, *I*_Na,L_, *I*_Ca,L_, and *I*_Kr_),	[110]
Doxapram	AF	II	Inhibition of K2P channels (TASK-1)	[117]
Bucindolol	AF in HF	I/II	Non-specific β-blocker α1-adrenoceptors blocker	[118]
Budiodarone	AF Patients with PM	II	Multichannel blockade	[113]
PKN605	AF	I	HDAC6 inhibitor	[114]

AF: Atrial fibrillation; HDAC6: histone deacetylase 6; K2P: family of two-pore domain K^+^ channels; PM: pacemaker; SVT: supraventricular tachycardia; TASK1: TWIK-related acid-sensitive potassium channel 1; TWIK: tandem of P-domains in a weakly inward rectifying K^+^ channel; VT: ventricular tachycardia.

### 4.8. Antiarrhythmic Agents and Pulmonary Vascular Disease

Overall, the antiarrhythmic pharmacological landscape is undergoing a renewed expansion driven by novel delivery routes, selective molecular targets, advanced ion-channel modulation, and in some cases, personalized therapeutic strategies. Although larger and longer studies are still required, these molecules constitute the most significant antiarrhythmic innovation of the past decade. While the role of antiarrhythmic therapies is primarily established in systemic cardiovascular disorders, their relevance in pulmonary vascular disease remains less well-defined. However, overlapping mechanisms related to autonomic regulation and myocardial remodeling may suggest potential areas for further investigation.

## 5. Therapies for Heart Failure

Heart failure (HF) currently represents one of the major challenges in CV medicine, due both to its high prevalence and its substantial clinical, economic, and societal impact. Recent epidemiological studies indicate that more than 6.7 million adults in the USA are living with HF, a figure expected to continue rising steadily over the coming decades, with estimates reaching 8.7 million by 2030 and more than 10 million by 2040. Moreover, the lifetime risk of developing HF is already approaching 24% of the adult population, reflecting an increasing burden particularly among younger individuals and those with multiple comorbidities. Mortality associated with HF remains high. Recent data indicate that HF in the USA contributed to more than 425,000 deaths in 2022, representing approximately 45% of all CV deaths, with a progressive increase observed since 2012 and a marked rise during 2020–2021. These trends underscore the need to strengthen preventive strategies and ensure comprehensive implementation of guideline-directed therapies [119,120].

Clinically, HF may manifest as either an acute or chronic condition, and its classification relies largely on the left ventricular ejection fraction (LVEF). Three phenotypes are recognized: heart failure with reduced ejection fraction (HFrEF, LVEF < 40%), heart failure with mildly reduced ejection fraction (HFmrEF), and heart failure with preserved ejection fraction (HFpEF, LVEF ≥ 50%). This heterogeneity reflects differences in underlying pathophysiological mechanisms, including neurohormonal dysregulation, chronic inflammation, and myocardial remodeling. Particularly, the prevalence of HFpEF continues to rise and is associated with one-year mortality rates of 20–29%, frequent hospitalization, and a substantial economic burden [121,122].

The treatment of HF has evolved markedly in recent decades. Following recognition of the role of peripheral vasodilation in the 1960s and 1970s, ACEi became established as foundational therapy. Subsequently, ARBs, BBs, and MRAs demonstrated significant benefits in reducing morbidity and mortality among patients with HFrEF. In recent years, additional therapeutic pillars have emerged, including sodium–glucose cotransporter-2 inhibitors (SGLT2i), now broadly recommended due to their beneficial effects on hospitalizations and mortality in HFrEF, as well as their usefulness in reducing decompensation-related admissions in HFpEF [123,124,125]. More recently, angiotensin receptor–neprilysin inhibitors (ARNi), represented by the combination sacubitril/valsartan, have become one of the most significant advances in HF therapy. ARNi simultaneously target the renin–angiotensin–aldosterone system and the natriuretic peptide pathway, thereby enhancing the cardioprotective effects of the latter. Their introduction has demonstrated clinical superiority over previous therapies in HFrEF, with reductions in CV mortality, rehospitalizations, and adverse ventricular remodeling [126,127].

Furthermore, recent advances have expanded diagnostic and therapeutic approaches, including improved recognition of specific cardiomyopathies such as amyloidosis and hypertrophic cardiomyopathy (HCM), as well as targeted strategies addressing conditions such as iron deficiency or relying on more precise device-based management. These developments, supported by updated international clinical practice guidelines, continue to reshape the therapeutic landscape of HF [123].

Collectively, these advances have enabled more refined stratification, diagnosis, and treatment of HF, with demonstrated improvements in quality of life, hospitalization rates, and survival. Table 5 summarizes the most relevant pharmacological treatments used in acute and chronic HF, particularly in patients with reduced ejection fraction.

In addition, pulmonary hypertension (PH) represents a closely related condition and a frequent complication among patients with HF, with prevalence rates reported as high as 80–83% regardless of LVEF phenotype. Sustained elevation of left ventricular filling pressures leads to retrograde transmission of pressure into the pulmonary vasculature, resulting in post-capillary PH. Over time, this hemodynamic overload can induce pulmonary vascular remodeling and right ventricular dysfunction, ultimately contributing to a worse prognosis and more complex clinical progression [128,129]. The coexistence of HF and PH exacerbates symptoms, increases hospitalization rates, and is associated with higher mortality. Optimized HF therapies, including SGLT2i and ARNi, have demonstrated hemodynamic improvement and potential benefit in the management of PH-HFpEF. This pathophysiological overlap highlights the need for rigorous hemodynamic assessment and individualized treatment strategies that prioritize optimal HF management as a central component of care [129].

Table 5 lists the pharmacological classes that include the main drugs indicated for the treatment of HF, which are discussed in some detail below. Table 6 provides a concise overview of certain drugs, used in various CV conditions, that in certain cases and conditions are indicated for HF treatment: specific forms of HF, to manage the symptoms or complications of HF, in conditions requiring combination therapy, or administered to patients who may not tolerate other medications.

### 5.1. Neurohormonal Modulation

HF is characterized by sustained activation of compensatory neurohormonal systems, primarily the RAAS, the sympathetic nervous system (SNS), and, to a lesser extent, the vasopressin system. Although initially adaptive, this chronic activation promotes vasoconstriction, sodium and water retention, ventricular remodeling, fibrosis, and apoptosis, thereby contributing to disease progression and a poor prognosis. Pharmacological inhibition of these pathways constitutes one of the central conceptual pillars of modern heart failure therapy [130].

*Angiotensin-Converting Enzyme Inhibitors (ACEi).* As noted in previous sections, ACEi block the conversion of angiotensin I to angiotensin II, thereby reducing vasoconstriction, aldosterone secretion, and sympathetic activation. In addition, they increase bradykinin levels by preventing its degradation, resulting in vasodilatory and antifibrotic effects. ACEi are first-line therapies for patients with HF and left ventricular dysfunction. They have been shown to improve survival in patients with chronic heart failure ranging from mild to severe. Classic trials in patients with HFrEF, such as CONSENSUS and SOLVD, which investigated the effects of enalapril, demonstrated significant reductions in mortality and hospitalizations, as well as attenuation of ventricular remodeling. Adverse effects include cough and angioedema due to bradykinin accumulation, hypotension, deterioration of renal function, and hyperkalemia; angioedema is infrequent but potentially life-threatening [131]. Several studies have shown that ACE inhibition across different patient populations (hypertensive individuals, patients with symptomatic HF, elderly patients with HF, and those with prior myocardial infarction) confers additional benefits such as regression of left ventricular hypertrophy, reduction of coronary vasoconstriction, anti-macrophage effects, stabilization of atherosclerotic plaques, and prevention of ventricular remodeling [132].

*Angiotensin II Receptor Blockers (ARBs).* ARBs selectively block the angiotensin II type 1 receptor (AT_1_), potentially providing a more complete blockade of angiotensin II effects. Indicated primarily in patients intolerant to ACEi, ARBs reduce hospitalizations and improve symptoms, although prognostic benefits are somewhat less consistent than those observed with ACEi when used as monotherapy. Their safety profile is similar to that of ACEi but with a lower incidence of cough and angioedema, as they exert minimal effects on bradykinin metabolism. However, their benefit may be offset by an increased risk of hyperkalemia [131,132].

*Mineralocorticoid Receptor Antagonists (MRAs).* By antagonizing the mineralocorticoid receptor, these agents inhibit aldosterone activity, thereby reducing myocardial and vascular fibrosis, inflammation, and sodium retention. The main MRAs are spironolactone and eplerenone. Clinical trials such as RALES (spironolactone 25 mg/day in patients with severe HF) and EMPHASIS-HF (eplerenone in patients with systolic HF and mild symptoms) demonstrated significant reductions in all-cause and CV mortality in HFrEF [133]. Their benefit extends to earlier stages of the disease. Although MRA are effective in HFrEF, safety considerations represent a limiting factor due to the risk of hyperkalemia and renal impairment; moreover, spironolactone may cause antiandrogenic endocrine effects, as it increases progesterone receptor expression and decreases androgen receptors [130,132]. In this regard, a recent meta-analysis of 32 clinical studies showed comparable efficacy between spironolactone and eplerenone; however, eplerenone demonstrated superior safety due to its greater selectivity, although it may still cause hyperkalemia. Finerenone was identified as the safest MRA, while canrenone showed considerable efficacy; nevertheless, data on these agents remain limited [134].

*Angiotensin Receptor–Neprilysin Inhibitors (ARNi).* This class currently comprises a single agent: the combination of the ARB valsartan with the neprilysin inhibitor sacubitril. In this formulation, AT_1_ receptor blockade (valsartan) is complemented by inhibition of neprilysin (sacubitril). Neprilysin is a peptidase responsible for the degradation of natriuretic peptides.

Three natriuretic peptides have been identified: atrial natriuretic peptide (ANP; type A), brain natriuretic peptide (BNP; type B), and type C natriuretic peptide (CNP). Only ANP and BNP are primarily secreted by cardiomyocytes. BNP is termed “brain” natriuretic peptide because it was initially discovered in porcine brain tissue. ANP and BNP are released in response to cardiac cell stress or stretch, which increases preload in the failing heart. These peptides activate natriuretic peptide receptor A, which in turn stimulates sGC, increasing intracellular cyclic guanosine monophosphate (cGMP). Through a cascade of phosphorylation and dephosphorylation events, cGMP reduces cytosolic calcium, leading to smooth muscle relaxation and vasodilation, which clinically translates into reduced afterload in the failing heart [135,136].

Proteolytic degradation of natriuretic peptides, mainly by neprilysin, removes these mediators from the circulation. Consequently, inhibition of neprilysin-mediated degradation has become an important therapeutic strategy in HF. Thus, the combination of sacubitril and valsartan enhances vasodilatory, natriuretic, and antifibrotic pathways while simultaneously inhibiting the RAAS, achieving dual neurohormonal modulation [127].

Among the major clinical studies evaluating sacubitril/valsartan, the PARADIGM-HF trial demonstrated superiority over enalapril in reducing CV mortality and HF hospitalizations in HFrEF [137]. Subsequent evidence and recent meta-analyses have confirmed improvements in clinical outcomes and quality of life in HFmrEF and, to a more modest extent, in HFpEF. Real-world data published in 2026 showed 10–25% reductions in all-cause and CV mortality, along with reverse ventricular remodeling [138,139]. Regarding safety, sacubitril/valsartan is associated with a higher incidence of symptomatic hypotension compared with ACEi or ARB, but with a lower risk of renal deterioration and severe hyperkalemia. A washout period after ACEi therapy is required to minimize the risk of angioedema [138].

*Beta-Blockers (BBs).* In chronic HF, there is persistent activation of the sympathetic nervous system as a compensatory response to reduced cardiac output. Although initially adaptive, this activation becomes maladaptive over time, promoting tachycardia, vasoconstriction, increased myocardial oxygen consumption, cardiomyocyte apoptosis, and adverse ventricular remodeling. Beta-blockers constitute one of the fundamental pillars of the pharmacological treatment of HF, particularly HFrEF. Their introduction represented a paradigm shift by demonstrating that sustained blockade of the sympathetic nervous system not only improves symptoms but also significantly modifies prognosis, reducing both mortality and HF-related hospitalizations [123,140,141].

As discussed in the section devoted to the use of beta-blockers in the treatment of hypertension, these agents act primarily through inhibition of β-adrenergic receptors, particularly β_1_ receptors, leading to reductions in heart rate and myocardial energy consumption, attenuation of catecholamine-mediated toxicity and cellular apoptosis, improvement in diastolic filling and ventricular mechanical efficiency, and, over the long term, partial reversal of adverse ventricular remodeling [142,143].

Robust scientific evidence supports the use of specific beta-blockers—carvedilol, bisoprolol, metoprolol succinate, and nebivolol—in patients with stable HFrEF. More than 20 years ago, large-scale randomized clinical trials demonstrated relative reductions in all-cause mortality of approximately 30–35% and significant decreases in HF-related hospitalizations [144,145,146,147]. These benefits are dose-dependent and are maintained over the long term, provided that therapeutic adherence is ensured [140,148]. In HFpEF, the role of beta-blockers is less well defined. Their use is mainly justified for heart rate control and for the treatment of comorbid conditions such as arterial hypertension, angina, or atrial fibrillation, rather than for a clearly demonstrated prognostic benefit [123,141,149].

The most frequent adverse effects include bradycardia, hypotension, fatigue, and, occasionally, transient worsening of HF symptoms during the initial titration phase. However, these effects are usually reversible with dose adjustment. Abrupt withdrawal is contraindicated, as it may precipitate acute decompensation or ischemic events. From a long-term safety perspective, beta-blockers exhibit a clearly favorable profile [123,140,141].

Within the framework of contemporary HF management, beta-blockers retain a central role as neurohormonal modulators and clearly prognosis-modifying therapies. Their integration into combined therapeutic strategies reflects the transition from a purely hemodynamic approach toward a mechanistic and multidimensional management of heart failure.

In summary, neurohormonal modulation represents the central axis of contemporary pharmacological treatment for HF, as it targets the fundamental pathological mechanisms that drive disease progression. Inhibition of the RAAS and the sympathetic nervous system, together with enhancement of natriuretic peptide pathways, has consistently been shown not only to improve symptoms but also to substantially modify prognosis, reducing mortality and hospitalizations and promoting reversal of ventricular remodeling. The rational integration of these therapies has marked the transition from a predominantly hemodynamic approach toward a mechanistic and multidimensional framework for HF management.

The future perspectives of neurohormonal pharmacotherapy point toward greater therapeutic precision, with increasingly personalized strategies that combine multiple biological targets, optimize safety, and extend benefits to HF phenotypes that were previously devoid of effective treatments. The development of new selective modulators, refinement of established therapies, and their integration with emerging approaches, including metabolic modulators, anti-inflammatory agents, and myocardial-targeted therapies, consolidate neurohormonal modulation not only as crucial in current management of HF but also as an essential platform upon which future therapeutic options are being built.

### 5.2. SGLT2 Inhibitors

Sodium–glucose cotransporter type 2 inhibitors (SGLT2i) represent one of the most significant advances in the pharmacotherapy of HF over the past decade. Initially developed as antidiabetic agents, their CV benefits became evident early in large CV safety trials and were subsequently consolidated in studies specifically designed for HF populations, demonstrating significant reductions in adverse CV events irrespective of the presence of diabetes mellitus. This paradigm shift has positioned SGLT2i as a central therapeutic class in the contemporary management of HF [150,151,152].

The SGLT2 cotransporter is predominantly expressed in the renal proximal tubule and is responsible for the reabsorption of approximately 90% of filtered glucose. Its inhibition induces glucosuria and natriuresis, resulting in a modest but sustained reduction in plasma volume and BP, with minimal activation of classical neurohormonal systems. These hemodynamic effects contribute to reductions in preload and afterload, improving congestion without compromising renal perfusion, which is particularly relevant in patients with advanced HF or concomitant renal disease [153,154].

Beyond their renal actions, numerous experimental and clinical studies have highlighted nonglycemic mechanisms of SGLT2i that are of particular relevance to the pathophysiology of HF. These include improved myocardial energetic efficiency through shifts in substrate utilization (increased oxidation of fatty acids and ketone bodies), optimization of mitochondrial function, enhancement of autophagy, and reductions in oxidative stress and myocardial inflammation. In addition, SGLT2i favorably modulate the Na^+^/H^+^ exchanger type 1, leading to reductions in cytosolic calcium and improved diastolic relaxation. Collectively, these effects place SGLT2i within a therapeutic paradigm that targets key cellular and metabolic processes involved in the progression of HF [155,156,157,158].

The clinical impact of SGLT2i in HF was robustly established by the DAPA-HF (dapagliflozin) and EMPEROR-Reduced (empagliflozin) trials, conducted in patients with HFrEF. Both studies demonstrated significant reductions in the composite endpoint of CV death and HF hospitalization, with consistent benefits in patients with and without diabetes. These findings confirmed that the cardioprotective effects of SGLT2i are largely independent of their glucose-lowering action [159,160]. Subsequently, the EMPEROR-Preserved and DELIVER trials extended these benefits to HFpEF and HFmrEF, marking a milestone as the first therapies to show solid clinical evidence in these phenotypes, which have traditionally been associated with high symptomatic burden and a lack of prognostic disease-modifying treatments [161,162]. Recent meta-analyses incorporating data from more than 100,000 patients confirm consistent reductions in HF hospitalizations and CV mortality across the entire ejection fraction continuum. In addition, real-world studies and long-term follow-up analyses suggest favorable effects on reverse ventricular remodeling, quality of life, and progression of chronic kidney disease, reinforcing the concept of integrated cardiorenal protection as a key mechanistic hallmark of this therapeutic class [158,163,164].

SGLT2i exhibit an interesting safety profile. The most common adverse events are genital mycotic infections, which are generally mild and preventable with appropriate hygiene measures. The risk of hypoglycemia is low in the absence of concomitant insulin or sulfonylurea therapy, and euglycemic ketoacidosis is infrequent in the HF setting. From a CV and renal standpoint, these agents are not associated with clinically relevant hypotension or significant acute renal deterioration; on the contrary, they slow the decline in glomerular filtration rate over the medium and long term [160,163,164].

SGLT2 inhibitors are regarded as a key first-line treatment in HFrEF alongside classical neurohormonal modulators, and one of the very few treatments with consistent prognostic evidence in HFpEF and HFmrEF. Their complementary mechanism of action positions them as a key component of integrated therapeutic strategies targeting hemodynamic, metabolic, and structural goals [158,161,162].

Although SGLT2i are not considered specific therapies for the treatment of pulmonary hypertension (PH), they exert several potentially relevant effects on the hemodynamic and mechanical determinants of the pulmonary circulation in HF [165,166]. Their natriuretic and osmotic diuretic actions sustainably reduce pulmonary venous congestion and left-sided filling pressures, which may translate into an indirect decrease in pulmonary capillary pressure and right ventricular afterload, with a more stable hemodynamic profile than that observed with conventional diuretics [167,168]. Moreover, experimental and clinical data suggest that SGLT2i exert favorable effects on right ventricular function and right ventricle–pulmonary artery coupling, a key axis in the progression of HF associated with PH.

Improvements in myocardial metabolic efficiency, reductions in oxidative stress, and modulation of intracellular ionic exchange may attenuate dysfunction of the pressure-overloaded right myocardium. In preclinical models, reductions in myocardial and vascular fibrosis have also been described, potentially limiting adverse remodeling of both the right ventricle and the pulmonary vasculature [169,170]. In clinical studies, although large trials of SGLT2i were not designed to directly evaluate invasive pulmonary hemodynamic parameters, secondary analyses and smaller mechanistic studies have demonstrated reductions in indirect markers of pulmonary pressure and right ventricular load. The consistency of clinical benefits observed in HF patients with comorbidities frequently associated with PH reinforces the hypothesis of a favorable impact on the cardiopulmonary axis [158,163,164].

From a conceptual perspective, SGLT2i may be viewed as modulators of the hemodynamic and metabolic milieu that reduce the mechanical burden transmitted to pulmonary circulation, rather than as direct pulmonary vasodilators. Their role may be particularly relevant in the early stages of PH associated with HF. Overall, although the available evidence does not justify the use of SGLT2i as targeted therapy for PH, their early integration into HF management may indirectly contribute to the prevention or attenuation of pulmonary hemodynamic load and its mechanical consequences on the right ventricle. Further studies with detailed hemodynamic characterization and specific PH criteria are required to more precisely define the impact of these agents across this cardiopulmonary continuum.

Finally, SGLT2 inhibitors exemplify a new generation of therapies with multifaceted mechanisms of action, including modulation of endothelial function, inflammation, myocardial energetics, and cardiorenal coupling. Notably, emerging evidence suggests a potential role in the regulation of sympathetic nervous system activity, a key driver of disease progression in cardiovascular disorders. Although data remain partly conflicting, both experimental and clinical studies indicate that SGLT2 inhibition may attenuate sympathetic overactivation without disrupting autonomic homeostasis [171]. These pleiotropic properties reinforce the concept that contemporary and emerging pharmacological strategies act on integrated biological networks rather than isolated targets, with potential implications not only for systemic cardiovascular disease but also for pulmonary vascular biology, where neurohormonal dysregulation, vascular remodeling, and metabolic stress converge.

### 5.3. Soluble Guanylate Cyclase (sGC) Stimulators

Cyclic guanosine monophosphate (cGMP) is a second messenger whose sustained intracellular increase activates multiple molecular effectors, including the coordinated phosphorylation of key targets in vascular smooth muscle cells and cardiomyocytes, such as L-type calcium channels, sarcoplasmic reticulum proteins, and cytoskeletal components. These events reduce cytosolic calcium levels, promote vascular relaxation, and improve ventricular distensibility. At the myocardial level, cGMP-mediated signaling also exerts anti-hypertrophic, anti-fibrotic, and anti-inflammatory effects by modulating pathways involved in adverse structural remodeling in chronic heart failure [172,173].

The pathophysiology of HF is characterized by an early and progressive disruption of the NO–sGC–cGMP signaling system, leading to a direct impairment of the physiological actions of cGMP. Indeed, oxidative stress, chronic inflammation, and endothelial dysfunction lead to reduced NO bioavailability, oxidation of the sGC heme moiety, and functional desensitization of the enzyme to endogenous nitrergic signaling. Consequently, cGMP generation and the activation of cGMP-dependent cytoprotective pathways are diminished, contributing to increased vascular tone, myocardial dysfunction, ventricular stiffness, and adverse remodeling [173,174].

It is therefore evident that sGC plays an essential role in this signaling pathway, as it catalyzes the conversion of guanosine triphosphate (GTP) to cGMP. From a structural perspective, sGC is a cytosolic heterodimer composed of α and β subunits, the latter containing a ferrous heme group (Fe^2+^) that is essential for NO binding. sGC stimulators bind to an allosteric site distinct from the heme moiety, stabilizing the enzyme in its active, reduced conformation. This mechanism allows for an increase in basal sGC activity independently of NO availability and, at the same time, enables a synergistic enhancement of the response to the low NO concentrations that persist in the setting of HF. Accordingly, sGC represents an important therapeutic target, and its activation and/or stimulation is beneficial in counteracting the pathophysiological effects of HF [172,175,176,177].

A key pharmacological distinction between sGC stimulators and NO-mobilizing or NO-donating agents, such as organic nitrates, is that sGC stimulators do not rely on metabolic bioactivation, do not induce significant hemodynamic tolerance, and do not promote uncontrolled generation of reactive nitrogen species. This molecular profile translates into a more physiological and sustained activation of cGMP signaling, with favorable effects on both acute hemodynamic variables (preload and afterload) and medium- to long-term cellular processes [173,175].

In clinical practice, vericiguat is currently the only approved sGC stimulator for the treatment of HFrEF. Its efficacy was demonstrated in the VICTORIA trial, which enrolled patients with recent worsening of HF despite optimized medical therapy. From a translational perspective, the observed benefits, primarily a reduction in HF-related hospitalizations, are interpreted as the clinical consequence of partial restoration of the NO–sGC–cGMP axis in a setting of marked neurohormonal activation and oxidative stress [176,178,179]. Subsequent analyses, including real-world evidence studies and pooled data from the VICTORIA and VICTOR trials, suggest that the therapeutic impact of sGC stimulation is greater in patients with high-risk molecular phenotypes. In contrast, in stable populations intensively treated with contemporary disease-modifying therapies, the incremental benefit appears more modest, supporting a strategy of biological and hemodynamic stratification to optimize patient selection [180].

**Table 5:** Summary of the main drugs with a clearly defined therapeutic indication for the treatment of heart failure.

Pharmacological Class	Representative Example	Mechanism and Effects	Clinical Use Indication	Refs.
ACEi	Enalapril	ACE inhibition RAAS blockade ↓ Vasoconstriction ↓ Na retention ↓ Ventricular remodeling	Standard HF treatment. ↓ Morbidity ↓ Mortality	[123]
ARB	Olmesartan/Valsartan	AT1 receptor antagonism RAAS blockade. ↓ Vasoconstriction ↓ Na retention	Standard HF treatment when ACEi not tolerated	
MRAs	Spironolactone	Aldosterone receptor antagonism ↓ Fibrosis ↓ Na retention ↓ Ventricular remodeling	HFrEF with prognostic benefit ↓ Morbidity ↓ Mortality	
ARNi	Sacubitril +Valsartan	Neprilysin inhibition + AT1 antagonism (RAAS blockade) ↑ Natriuretic peptides ↓ Ventricular remodeling	First-line in HFrEF; ↓ CV mortality and hospitalizations	[126,127,181]
Beta- blockers	Carvedilol/Bisoprolol/Metoprolol/ Nebivolol	β1-receptor blockade ↓ Harth rate ↓ Myocardial O_2_ demand; ↓ Ventricular remodeling	Improves clinical outcomes ↓ hospitalizations ↓ mortality *Not all BB are indicated for HF*	[123,148]
SGLT2i	Dapagliflozin/Empagliflozin	Renal SGLT2 inhibition ↓ Renal reabsorption of glucose & Na ↑ Glucosuria ↓ Plasma volume Improved hemodynamics Anti-inflammatory effects	Indicated in HFrEF and HFpEF ↓ CV mortality and hospitalizations	[123,125]
sGC stimulators	Vericiguat	Activation of sGC ↑ cGMP ↑ Pulmonary vasodilation Hemodynamic improvement	Vericiguat indicated in HFrEF PH and CTEPH. In HFpEF with PH	[129,176,182]

ACE: Angiotensin-converting enzyme; ACEi: angiotensin-converting enzyme inhibitor; ARB: angiotensin II receptor blocker; ARNi: angiotensin receptor–neprilysin inhibitors; AT1: angiotensin II receptor type 1; BB: β-adrenergic receptor blocker; CTEPH: chronic thromboembolic pulmonary hypertension; CV: cardiovascular; HF: heart failure; HFpEF: heart failure with preserved ejection fraction; HFrEF: heart failure with reduced ejection fraction; MRA: mineralocorticoid receptor antagonists; PH: pulmonary hypertension; RAAS: renin–angiotensin–aldosterone system; SGLT2i: sodium–glucose cotransporter 2 inhibitors. ↑ means an increase in levels and ↓ means a decrease.

Finally, other modulators of this system, such as sGC activators, which are capable of targeting the enzyme in its oxidized or heme-free form, have demonstrated pronounced hemodynamic effects under conditions of severe oxidative stress, but have not yet shown a clear clinical benefit in chronic HF. Nevertheless, these compounds remain of interest as pharmacological tools to explore specific patient subgroups and to further elucidate the mechanistic role of the NO–sGC–cGMP sequence as a CV therapeutic target [174,175,177].

### 5.4. Vasodilators/NO Pathway

Vasodilation represents a fundamental therapeutic target in HF, as it enables reductions in preload and afterload, improves cardiac output, and alleviates pulmonary and systemic congestion. Within this context, the NO pathway constitutes one of the principal endogenous regulatory systems of vascular tone and myocardial function. However, in chronic HF, this pathway is frequently disrupted as a consequence of endothelial dysfunction, oxidative stress, and sustained neurohormonal activation. For this reason, vasodilation through modulation of NO signaling has historically been a priority therapeutic target, not only because of its ability to regulate vascular tone but also owing to its effects on cardiac remodeling, inflammation, and endothelial function [183,184].

NO is produced primarily by endothelial nitric oxide synthase (eNOS) and, as discussed in previous sections, exerts its effects through activation of sGC, leading to increased intracellular cGMP levels and resulting in vasodilation, reduction of ventricular afterload, improved vascular compliance, and antifibrotic and anti-inflammatory effects [173].

Organic nitrates, such as nitroglycerin, isosorbide dinitrate, and isosorbide mononitrate, constitute the classical vasodilators that modulate NO release, thereby increasing cGMP production in vascular smooth muscle through indirect activation of sGC. Their predominant hemodynamic effect is venous vasodilation, resulting in a marked reduction in preload, which lowers pulmonary capillary pressure and rapidly relieves congestion. At higher doses, they also induce arterial vasodilation and reduce afterload. In clinical practice, nitrates are used mainly in acute or decompensated HF, where their rapid effects on ventricular hemodynamics effectively improve symptoms and hemodynamic parameters. However, in chronic HF, endothelial NO production via eNOS is frequently impaired, and, together with the rapid inactivation of NO by reactive oxygen species, this leads to functional uncoupling of NO-dependent signaling. This phenomenon limits the efficacy of classical NO donors and helps explain the lack of sustained benefit of nitrates as chronic monotherapy [185,186,187].

Hydralazine is a direct arterial vasodilator which, although it does not act as an NO donor, reduces systemic vascular resistance and attenuates oxidative stress, thereby contributing to preservation of endogenous NO bioavailability. The combination of hydralazine with isosorbide dinitrate represents a classical pharmacological approach that is nevertheless well grounded in pathophysiology, as it has demonstrated benefits in terms of reduced mortality and hospitalizations in patients with HFrEF by coupling preload and afterload reduction with enhancement of NO signaling. From a hemodynamic standpoint, this combination simultaneously reduces afterload and preload, improving stroke volume and decreasing ventricular workload. Mechanistically, it may partially counteract endothelial dysfunction and reduced NO bioavailability, which could explain its benefit in populations with marked vascular impairment or intolerance to RAAS inhibitors. Accordingly, this therapeutic combination continues to be recommended in patients who cannot tolerate RAAS inhibitors or as add-on therapy in selected populations [186,187,188].

Beyond its effects on vascular tone, NO modulates several processes relevant to HF progression, including myocardial hypertrophy, ventricular stiffness, platelet aggregation, and vascular inflammation. Disruption of this signaling pathway therefore has both hemodynamic and structural consequences, reinforcing the relevance of the NO pathway as a therapeutic target, despite the limitations of currently available pharmacological agents [189].

Overall, classical vasodilators and direct NO modulators have lost prominence as first-line therapies and now play a more selective and context-dependent role, particularly in settings of acute decompensation, intolerance to other prognosis-modifying treatments, or specific hemodynamic profiles. Nonetheless, their conceptual relevance remains central, as from a mechanistic perspective these agents remain valuable tools for understanding the interplay between ventricular loading conditions, vascular function, and molecular signaling—key elements in an integrated approach to the hemodynamic and mechanical targets of heart failure.

The NO–sGC–cGMP pathway thus constitutes an integrative axis that links traditional hemodynamic strategies with contemporary therapies based on molecular mechanisms. It provides a shared pathophysiological framework that explains part of the observed benefits of ARNIs, SGLT2 inhibitors, and other emerging therapies targeting vascular and metabolic pathways.

### 5.5. Inotropes/Inodilators

Systolic dysfunction and reduced cardiac output are central components in the pathophysiology of HF, particularly in its acute, advanced, or complicated forms associated with systemic hypoperfusion. In this context, inotropes and inodilators have been used with the aim of increasing myocardial contractility, improving stroke volume, and restoring adequate tissue perfusion. In contrast to prognostic disease-modifying therapies employed in stable chronic HF, these agents are used primarily as hemodynamic supportive therapy, with specific indications and generally limited durations of administration [190,191,192,193]. Classic inotropic agents can be grouped into three major categories based on their mechanism of action: β-adrenergic agonists, phosphodiesterase type 3 (PDE3) inhibitors, and calcium sensitizers.

β-Adrenergic agonists, such as dobutamine, enhance contractility through β1-receptor activation and increased intracellular cyclic AMP (cAMP), which augments Ca^2+^ influx into the cardiomyocyte. Although they produce a rapid increase in cardiac output, this mechanism is associated with increased myocardial oxygen consumption and a higher risk of arrhythmias [192,194].

PDE3 inhibitors, such as milrinone, prevent the degradation of cAMP in both cardiomyocytes and vascular smooth muscle, thereby combining a positive inotropic effect with arterial and venous vasodilation, which justifies their classification as inodilators. Unlike β-agonists, their effects are independent of adrenergic receptors, a feature that may be advantageous in patients receiving chronic β-blocker therapy. However, their use is associated with risks of hypotension, arrhythmias, and worsening renal function [190,193].

Calcium sensitizers, primarily represented by levosimendan, increase contractility by stabilizing the calcium–troponin C interaction without increasing intracellular Ca^2+^ concentrations, thereby improving systolic function with a lesser impact on myocardial energy consumption. In addition, levosimendan opens ATP-dependent K^+^ channels in vascular smooth muscle, producing systemic and pulmonary vasodilation, as well as potential cardioprotective effects at the mitochondrial level [195].

The main clinical setting for the use of inotropes and inodilators is acute HF with low cardiac output and signs of hypoperfusion, including cardiogenic shock and severe decompensation of advanced HF. In these scenarios, numerous studies have demonstrated transient improvements in hemodynamic parameters, such as cardiac output and perfusion pressure, as well as rapid symptom relief [190,191]. However, available clinical trials and meta-analyses have not consistently demonstrated a clear survival benefit with prolonged use of classic inotropes, and some have even suggested a potential association with increased mortality when these agents are used chronically or without careful selection. This observation has led clinical guidelines to recommend their use at the lowest effective dose and for the shortest possible duration, reserving them for situations of hemodynamic instability or as a bridge to definitive therapies such as ventricular assist devices or heart transplantation [192,194,196].

Levosimendan has attracted particular interest as an inodilator because of its distinct mechanistic profile. Studies and meta-analyses suggest that intermittent administration of levosimendan in patients with advanced HF may improve left ventricular ejection fraction, reduce natriuretic peptide levels, and, in some analyses, decrease mortality, without a clear increase in serious adverse events. Nevertheless, these benefits have not been uniformly observed across all trials, and its impact on hospitalization rates remains a matter of debate [195,197].

From a safety perspective, the main concerns associated with inotropes are the risks of ventricular arrhythmias, myocardial ischemia, hypotension, and increased oxygen consumption, particularly with agents that act by increasing intracellular Ca^2+^ concentrations. These adverse effects partly explain the poorer prognosis observed with prolonged use and have prompted an increasingly restrictive and individualized therapeutic strategy [192,193,196]. In the case of inodilators, hypotension and renal dysfunction represent the most relevant complications, especially in patients with reduced intravascular volume or pre-existing renal impairment. Consequently, their use requires close hemodynamic monitoring and careful patient selection [195].

At present, inotropes and inodilators are no longer considered therapies intended to modify the natural history of HF, but rather hemodynamic support tools useful in well-defined clinical scenarios. From a mechanistic perspective, however, they have contributed crucially to the understanding of the relationship between contractility, ventricular loading conditions, energy consumption, and systemic perfusion. This knowledge has driven the development of new therapeutic strategies, such as cardiac myosin activators and metabolic modulators, which aim to improve cardiac function with more favorable safety profiles [198].

**Table 6:** Some drugs, used in different cardiovascular conditions, indicated, in certain cases and conditions, for the treatment of HF: specific forms of HF, to manage the symptoms or complications of HF, in conditions requiring combination therapy, or administered to patients who may not tolerate other medications.

Pharmacological Class	Representative Example	Mechanism and Effects	Clinical Use Indication	Refs.
Nitrates	Isosorbide dinitrate	NO donor Venodilation ↓ Preload ↓ Pulmonary congestion	Symptom relief in HFrEF Often combined with hydralazine	[199,200]
Direct Vasodilators	Hydralazine	Relaxes arteriolar smooth muscle via NO/sGC signaling ↓ Systemic vascular resistance	HFrEF in patients intolerant to ACEi/ARB	
Thiazide Diuretics	Hydrochlorothiazide	Inhibits the Na^+^/Cl^−^ cotransporter in the distal convoluted tubule ↑ Natriuresis ↓ Circulating volume ↓ Preload	Acute decompensated HF Edema associated with congestive HF	
Inotropes/ Inodilators	Dobutamine Milrinone Levosimendan	β_1_ agonist PDE3 inhibitor Calcium sensitizer ↑ Contractility ↑ Cardiac output ↑ Arrhythmias ↑ Hypotension Renal effects	Acute decompensated HF with low cardiac output and signs of hypoperfusion	[192,193,197]
Ca^2+^ Channel Blockers	Amlodipine	Blocks L-type Ca^2+^ channels ↓ Vascular resistance ↓ Afterload No direct improvement in cardiac contractility	Used in hypertension/angina Not recommended in HFrEF May be useful in HFpEF for BP control	[13,201]
Antiarrhythmics	Digoxin	Inhibits Na^+^/K^+^ ATPase ↑ Intracellular Ca^2+^ Positive inotropic effect	Rate control in AF Symptom control in HFrEF	
ERA	Bosentan	Antagonizes endothelin A/B receptors ↓ Pulmonary vasoconstriction	HF secondary PH	[202]
PDE5 Inhibitors	Sildenafil	Inhibits PDE5 ↑ cGMP ↑ Pulmonary vasodilation	PH Not indicated in HFrEF	
Prostanoids	Iloprost	Prostacyclin agonism ↑ Pulmonary vasodilation e	PH	
Vasopressors	Norepinephrine/Vasopressin	α_1_-mediated vasoconstriction ↑ Systemic vascular resistance	Cardiogenic shock Not indicated for chronic HF Use only in cases of severe decompensation	[203]

ACEi: Angiotensin-converting enzyme inhibitor; AF: atrial fibrillation; ARB: angiotensin II receptor blocker; ERA: endothelin receptor antagonist; cGMP: cyclic guanosine monophosphate; HF: heart failure; HFpEF: heart failure with preserved ejection fraction; HFrEF: heart failure with reduced ejection fraction; PH: pulmonary hypertension; PDE3: phosphodiesterase type 3; PDE5: phosphodiesterase type 5; sGC: soluble guanylate cyclase. ↑ means an increase in levels and ↓ means a decrease.

### 5.6. Emerging and Future Pharmacological Targets in Heart Failure

Pharmacological development in HF reflects a progressive transition from predominantly hemodynamic modulation toward interventions targeting pathogenic mechanisms and phenotype-based patient stratification. This evolution is particularly relevant in clinical settings with unmet medical needs, such as HFpEF, HFmrEF, and right-sided HF associated with PH (Table 7).

One active line of research focuses on the direct modulation of myocardial contractility without increasing intracellular Ca^2+^, with the aim of improving systolic function while minimizing arrhythmogenic risk. Selective myosin activators represent a paradigmatic example of this approach. Omecamtiv mecarbil, the first agent in this class, demonstrated a modest reduction in HF-related events in patients with HFrEF in the phase III GALACTIC-HF trial, with an overall neutral safety profile; however, its subsequent clinical development has been limited. Next-generation myosin activators, such as danicamtiv, aim to preserve the benefits on systolic function while improving tolerability [198,204].

Another therapeutic approach targets fibrosis and inflammation, key processes in the pathophysiology of HFpEF and HF associated with cardiometabolic disorders. The non-steroidal MRA finerenone has demonstrated a favorable cardiorenal profile and antifibrotic effects, with ongoing phase III studies evaluating its potential role in HFpEF and HFmrEF [205]. Additional antifibrotic strategies include interference with profibrotic signaling pathways, such as connective tissue growth factor (CTGF) and transforming growth factor beta (TGF-β), using agents such as pirfenidone and pamrevlumab. Although early studies have provided promising mechanistic signals, their clinical efficacy in HF remains, to date, exploratory [206,207].

Pulmonary vascular remodeling and right ventricular (RV) dysfunction represent a critical point of convergence between CV and pulmonary disease. In this context, sotatercept has emerged as the first clearly disease-modifying therapy for pulmonary arterial hypertension (PAH). Sotatercept acts as a “ligand trap” that modulates signaling mediated by the BMP/TGF-β superfamily, a group of signaling proteins that regulate cellular proliferation, differentiation, and development, including activins and bone morphogenetic proteins (BMPs). Sotatercept has demonstrated substantial improvements in pulmonary hemodynamics and functional capacity. Its approval in 2024 for the treatment of PAH represents a major milestone with relevant implications for right-sided HF secondary to pulmonary vascular disease [208,209,210]. Other antiproliferative strategies, such as inhaled tyrosine kinase (TK) inhibitors including seralutinib and imatinib, seek to maximize pulmonary vascular effects while limiting systemic toxicity [211,212].

**Table 7:** Pharmacological compounds in clinical development for heart failure and related cardiovascular conditions.

Compound	Class/Mechanism	Status	Potential Indication	Efficacy and Safety	Refs.
Danicamtiv	Selective cardiac myosin activator Improves contractility	Phase IIb	HFrEF	Improves systolic function Acceptable tolerability Monitoring for myocardial ischemia recommended	[204,213]
Omecamtiv mercabil	Cardiac myosin activator Prolongs systolic ejection	Phase III GALACTIC-HF	HFrEF	Modest reduction in HF Neutral safety profile	[198,214]
Finerenone	Non-steroidal MRA	Phase III	HFpEF, HFmrEF Potential role in PH-LHD	↓ Myocardial fibrosis ↓ Inflammation Lower risk of hyperkalemia vs. with classical MRAs	[205]
Pamrevlumab	Anti-CTGF MAb Antifibrotic	Phase II	HFpEF fibrotic cardiomyopathy	↓ Fibrosis in experimental models Acceptable safety Clinical efficacy exploratory	[204,206]
Pirfenidone	Targets TGF-β signaling Collagen synthesis Antifibrotic agent	Phase II	HFpEF	Signals of reduced myocardial fibrosis Tolerability profile well characterized	[204,206]
Sotatercept Recombinant fusion protein	Traps activin A & GDF ligands Recalibrates TGF-β signaling	Approved for PAH (2024) Studies in right ventricular dysfunction	PAH Right-sided heart failure	Improvement in pulmonary hemodynamics & functional capacity Monitoring for erythrocytosis & systemic hypertension required	[208,210,212]
Seralutinib (inhaled)	TK inhibitor Anti-proliferative	Phase II (PAH)	PAH Right-sided heart failure	Improves pulmonary vascular resistance Inhaled administration reduces systemic toxicity	[211,212]
Imatinib (inhaled)	TK inhibitor Anti-proliferative	Phase II	Severe PAH	Improves pulmonary hemodynamics Favorable safety profile vs. oral administration	[212]

Abbreviations: CTGF: connective tissue growth factor; GDF: growth differentiating factor; HFrEF, heart failure with reduced ejection fraction; HFpEF, heart failure with preserved ejection fraction; HFmrEF, heart failure with mildly reduced ejection fraction; MAb: monoclonal antibody; MRA: mineralocorticoid receptor antagonist; PAH, pulmonary arterial hypertension; PH-LHD, pulmonary hypertension due to left heart disease; TGF: transforming growth factor: TK: tyrosine kinase. ↓ means a decrease in levels.

Several preclinical and early translational programs aim to correct fundamental cellular and molecular alterations in HF (Table 8). These include next-generation gene therapy vectors designed to restore SERCA2a (Sarcoplasmic/endoplasmic reticulum Ca^2+^ ATPase 2a) function. SERCA2a is an essential protein for cardiac muscle contraction and relaxation that plays a central role in cytosolic Ca^2+^ regulation by actively recycling cytoplasmic calcium; its dysfunction or downregulation is implicated in the pathogenesis of HF. Other strategies target intracellular Ca^2+^ handling, mitochondrial modulators aimed at improving myocardial bioenergetics, regenerative approaches using stem cells or CV exosomes, and RNA-based therapies with antifibrotic effects. The latter strategy uses microRNA (miRNA), which are small (21–23 nucleotides), single-stranded, non-coding RNA molecules involved in RNA silencing and post-transcriptional regulation of gene expression. Despite their strong conceptual appeal, these strategies face significant challenges related to delivery, patient selection, and the demonstration of a reproducible clinical benefit [204,206,207,215].

### 5.7. Heart Failure and Pulmonary Vascular Disease

Taken together, this landscape highlights a strategic transition toward precision CV pharmacology, in which deep phenotyping, identification of molecular targets, and alignment with disease mechanisms play a central role. HFpEF and PH secondary to left heart disease remain priority areas for innovation, reflecting both their biological complexity and the persistent lack of effective therapies. The translation of these emerging approaches into tangible clinical improvements will depend on rigorous trial design, the use of biomarkers for patient stratification, and a comprehensive assessment of long-term safety. In this context, these mechanisms, extensively characterized in heart failure, are also highly relevant to pulmonary vascular disease and right ventricular dysfunction, highlighting a shared pathophysiological framework that supports the translational potential of established and emerging therapies across vascular territories.

## 6. Therapies for Hypertrophic Cardiomyopathy

Hypertrophic cardiomyopathy (HCM) is the most common inherited cardiac disease, with an estimated prevalence of approximately 1 in 500 individuals worldwide. It is primarily caused by pathogenic variants in genes encoding sarcomeric proteins, particularly β-myosin heavy chain and myosin-binding protein C (MYBPC3), leading to myocardial hypercontractility, hypertrophy, impaired relaxation, and myocardial fibrosis. Clinically, HCM is characterized by marked phenotypic heterogeneity, ranging from asymptomatic disease to advanced HF, atrial and ventricular arrhythmias, and sudden cardiac death [216,217,218].

From a hemodynamic standpoint, HCM can be broadly classified into obstructive and non-obstructive forms, depending on the presence of dynamic left ventricular outflow tract obstruction (LVOTO). Traditional therapeutic strategies have primarily focused on symptom relief, reduction of LVOTO, and prevention of complications, rather than on modification of the underlying disease mechanisms [219,220].

### 6.1. Current Pharmacological Treatment

Conventional pharmacological management of symptomatic HCM is based on agents with negative inotropic effects, aimed at reducing myocardial contractility, prolonging diastolic filling, and alleviating dynamic obstruction. β-blockers are recommended as first-line therapy, particularly in patients with obstructive physiology, due to their ability to reduce heart rate and myocardial oxygen consumption. Non-dihydropyridine calcium channel blockers (verapamil and diltiazem) represent commonly used alternatives, especially in patients who do not tolerate β-blockers, although caution is required in the presence of severe obstruction or hypotension [221,222].

In selected patients with persistent symptoms, disopyramide, a class Ia antiarrhythmic agent with negative inotropic properties, may be added to further reduce left ventricular outflow tract obstruction. However, its anticholinergic adverse effects and proarrhythmic potential limit its widespread use. Importantly, these established therapies primarily act on downstream hemodynamic consequences and do not correct the sarcomeric dysfunction that lies at the core of HCM pathogenesis [221,222].

### 6.2. Cardiac Myosin Inhibitors

Cardiac β-myosin, a fibrous motor protein, is the main component of the thick filament in the human ventricular sarcomere and is responsible for contractility by sliding along actin filaments using ATP hydrolysis. Under physiological conditions, a substantial fraction of myosin molecules resides in an autoinhibited, low-energy-consumption super-relaxed state (SRX). Many HCM-associated mutations reduce the proportion of inhibited myosin and increase its availability to interact with actin, thereby enhancing basal contractility. This results in increased sarcomere tension even during diastole, elevated ATP consumption, energetic stress, and impaired Ca^2+^-dependent relaxation [223,224,225,226].

The introduction of cardiac myosin inhibitors (CMi) has represented a profound conceptual shift in the treatment of HCM, as they constitute the first disease-specific pharmacological approach targeting the fundamental molecular abnormality of sarcomeric hypercontractility. These agents act by reversibly inhibiting myosin ATPase activity, reducing excessive actin–myosin cross-bridge formation and normalizing myocardial contractile mechanics and energetic efficiency [227,228].

Mavacamten, the first-in-class CMi, has demonstrated robust clinical efficacy in patients with symptomatic obstructive HCM. In phase 2 and 3 clinical trials, mavacamten significantly reduced resting and provocable LVOTO gradients, improved exercise capacity and functional class, and was associated with clinically meaningful improvements in patient-reported quality of life [229,230]. In addition, multimodality imaging studies have provided evidence of favorable cardiac remodeling, including reductions in left ventricular mass and left atrial size. Long-term extension studies and real-world data indicate that these benefits are sustained over time, provided that careful dose titration and close echocardiographic monitoring are implemented to minimize the risk of reversible reductions in left ventricular ejection fraction [227,230,231].

Although preliminary improvements in biomarkers and symptoms have been observed in non-obstructive HCM, definitive clinical benefit in this population has not yet been clearly established [232].

Aficamten, a second-generation CMi with a shorter half-life and a more predictable pharmacokinetic profile, has shown comparable efficacy in reducing obstruction and improving symptoms in clinical trials and is currently in advanced stages of clinical development and regulatory review [228,233,234].

### 6.3. Emerging and Future Therapies

Beyond myosin inhibition, a wide range of emerging therapeutic strategies aim to address the genetic, molecular, and cellular determinants of HCM.

Cardiac myosin-binding protein C (MYBPC3) accounts for a substantial proportion of inherited HCM cases. It is a key regulatory protein localized to specific bands of the thick filament, where it modulates actin–myosin interactions and synchronizes contractile activation with phosphorylation status and β-adrenergic signaling, playing an active role in stabilizing the myosin SRX state. Consequently, pathogenic MYBPC3 variants lead to reduced protein functionality and impaired sarcomeric regulation.

Among the most advanced approaches are gene replacement therapies targeting MYBPC3. These consist of adeno-associated virus (AAV)-mediated delivery of functional copies of the MYBPC3 gene, which have been shown to reverse the pathological phenotype in preclinical models, with early-phase clinical trials currently underway in humans [235,236].

Other investigational approaches include allele-specific gene silencing, RNA-based therapies, and genome-editing technologies such as CRISPR-Cas9, aimed at directly correcting pathogenic variants. Despite their transformative potential, clinical translation faces significant challenges related to delivery efficiency, long-term safety, off-target effects, and ethical and equity considerations regarding access [237].

Complementary strategies are also being explored, focusing on modulation of myocardial bioenergetics, inhibition of profibrotic pathways, and the development of precision medicine approaches, integrating deep phenotyping, advanced imaging techniques, and circulating biomarkers to tailor therapy to the dominant disease mechanisms in individual patients [220,238].

Overall, the therapeutic management of hypertrophic cardiomyopathy is undergoing a fundamental transition from empiric, hemodynamics-based care toward mechanism-targeted and potentially disease-modifying pharmacology. Cardiac myosin inhibitors have already redefined the treatment of obstructive HCM, while gene- and RNA-based therapies open the possibility of definitive interventions in selected genetic subtypes. The successful integration of these innovations into clinical practice will depend on rigorous trial design, comprehensive evaluation of long-term safety, and a multidisciplinary approach, fully aligned with the vision of precision cardiovascular pharmacotherapy outlined in this review.

### 6.4. Hypertrophic Cardiomyopathy and Pulmonary Vascular Disease

Hypertrophic cardiomyopathy provides a paradigmatic example of how primary myocardial abnormalities can converge with broader cardiovascular and pulmonary vascular pathophysiology through shared hemodynamic, neurohormonal, and cellular mechanisms. Altered myocardial relaxation, increased filling pressures, and secondary neurohormonal activation in HCM may contribute to pulmonary vascular remodeling and elevated pulmonary pressures, linking this condition to the pathobiology of pulmonary hypertension and right ventricular dysfunction. Moreover, several therapeutic strategies reviewed in this section—including modulation of contractility, improvement of myocardial energetics, and emerging antifibrotic or gene-targeted approaches—target pathways that are also relevant to vascular tone regulation, endothelial function, and cellular proliferation within the pulmonary circulation. This overlap underscores the importance of considering HCM not only as a localized cardiomyopathic process but also within a broader cardiopulmonary continuum, in which mechanistic insights and therapeutic innovations may have translational implications across systemic and pulmonary vascular diseases.

## 7. Conclusions

Cardiovascular diseases remain the leading cause of global morbidity and mortality, and their clinical complexity continues to increase as patient populations age and comorbidities accumulate. Despite important therapeutic advances, optimal long-term control of cardiovascular risk and disease progression remains challenging. This review highlights that many apparently distinct cardiovascular conditions, such as hypertension, heart failure, arrhythmias, and hypertrophic cardiomyopathy, are driven by convergent disturbances in hemodynamic load, neurohormonal activation, electrophysiological regulation, and myocardial function.

Importantly, these shared mechanistic axes substantially overlap with the pathophysiology of pulmonary vascular disease, where dysregulation of vascular tone, endothelial signaling, neurohormonal pathways, and right ventricular loading plays a central role. Recognizing this convergence provides a conceptual framework for understanding disease progression across vascular beds and supports the rationale for translational pharmacological strategies that extend beyond traditional disease boundaries.

In this context, established pharmacological therapies targeting the renin–angiotensin–aldosterone system, sympathetic nervous system, calcium handling, electrophysiological conduction, and vascular tone remain the foundation of cardiovascular disease management and have demonstrated clear benefits in reducing morbidity and improving long-term outcomes. However, despite optimal use of these drug classes, many patients continue to experience residual risk, progressive disease, or suboptimal control of hemodynamic load, neurohormonal activation, and arrhythmic vulnerability. This therapeutic gap is particularly evident in patients with resistant disease, advanced cardiac dysfunction, or complex comorbidity profiles, underscoring the need for mechanism-guided strategies that extend beyond conventional phenotype-based treatment algorithms.

Recent years have witnessed significant advances in mechanism-based therapeutic approaches aimed at more selectively targeting disease-driving pathways, including SGLT2 inhibitors, soluble guanylate cyclase stimulators, endothelin receptor antagonists, myosin inhibitors, and related agents. In parallel, emerging strategies directed at aldosterone synthase inhibition, RNA interference-mediated modulation of the renin–angiotensin system, and aminopeptidase A blockade illustrate a broader shift toward upstream and biologically precise intervention. Collectively, these developments reflect an increasing focus on shared regulatory mechanisms that operate across cardiovascular phenotypes and vascular territories, opening new opportunities for translational and individualized pharmacological approaches.

Overall, continued innovation in cardiovascular pharmacotherapy is essential to address persisting unmet clinical needs. By prioritizing shared mechanistic pathways over isolated disease entities, emerging therapies have the potential to achieve more durable stabilization of hemodynamic load, neurohormonal activity, and electrophysiological disturbances. Importantly, this mechanism-guided perspective supports a transition toward more precise and individualized pharmacological strategies and may yield translational insights relevant not only to systemic cardiovascular disorders but also to diseases affecting the pulmonary vasculature. Collectively, the recognition of shared mechanistic pathways across systemic cardiovascular and pulmonary vascular diseases provides a unifying framework that may facilitate the development of more integrated and mechanism-driven therapeutic strategies.

## Figures and Tables

**Table 8:** Some preclinical programs of emerging therapeutic interest for heart failure.

Compound	Class Mechanism	Potential Indication	Efficacy and Safety	Refs.
SERCA2a gene therapy (next-generation AAV)	Enhancement sarcoplasmic Ca^2+^ reuptake	HFrEF	Proof of concept established; newer, more selective vectors developed after CUPID trial failure	[204,207]
Mitochondrial modulators (e.g., elamipretide-like)	Improvement bioenergetic efficiency	HFrEF HFpEF	Potential benefit in metabolic HF; prior clinical results inconsistent	[204,215]
RNA-based therapies (antifibrotic miRNA)	Epigenetic regulation of fibrosis	HFpEF PH-LHD	Highly specific approach; challenges related to delivery and safety	[204,207]
Cardiovascular stem cells/exosomes	Paracrine-mediated repair	Advanced HF	Regenerative signaling observed; lack of standardization and robust clinical endpoints	[206,207]

Abbreviations: AAV: adeno-associated viral vectors; HFrEF: heart failure with reduced ejection fraction; HFpEF: heart failure with preserved ejection fraction; miRNA: micro ribonucleic acid; PH-LHD: pulmonary hypertension secondary to left heart disease; SERCA2a: sarcoplasmic/endoplasmic reticulum Ca^2+^ ATPase 2a.

## Data Availability

The original contributions presented in this study are included in the article. Further inquiries can be directed to the corresponding authors.

## Abbreviations

AAD	Antiarrhythmic drug
ACE	Angiotensin converting enzyme
ACEi	Angiotensin converting enzyme inhibitors
AP	Action potential
AF	Atrial fibrillation
ANP	Atrial natriuretic peptide
APA-A	Aminopeptidase A
ARB	Angiotensin II receptor blocker. Angiotensin II receptor antagonist
ARNi	Angiotensin receptor–neprilysin inhibitors
AT_1_	Angiotensin II receptor type 1
ATP	Adenosine triphosphate
AV	Atrioventricular
BB	β-adrenergic receptor blocker; β-adrenergic receptor antagonist; beta blocker
BMP	Bone morphogenetic proteins
BNP	Brain natriuretic peptide
BP	Blood pressure
cAMP	Cyclic AMP
CCB	Calcium channel blocker
cGMP	Cyclic guanosine monophosphate
CMi	Cardiac myosin inhibitor
CNP	c-type natriuretic peptide
CTGF	Connective tissue growth factor
CV	Cardiovascular
CVD	Cardiovascular disease
CTEPH	Chronic thromboembolic pulmonary hypertension
eGFR	Estimated Glomerular Filtration Rate
ENaC	Epithelial sodium channel
eNOS	Endothelial nitric oxide synthase
ER	Endothelin receptor
ERA	Endothelin receptor antagonist
ESH	European Society of Hypertension
ET	Endothelin
ETR	Endothelin receptor
FDA	US Food & Drug Administration
GDF	Growth differentiating factor
GTP	Guanosine triphosphate
HCM	Hypertrophic cardiomyopathy
HDAC6	Histone deacetylase 6
HF	Heart failure
HFmrEF	Heart failure with mildly reduced ejection fraction
HFpEF	Heart failure with preserved ejection fraction
HFrEF	Heart failure with reduced ejection fraction
HCN	Hyperpolarization-activated cyclic nucleotide gated
K2P	Family of two-pore domain K^+^ channels
LV	Left ventricular
LVEF	Left ventricular ejection fraction
LVOTO	Left ventricular outflow tract obstruction
MAb	Monoclonal antibody
miRNA	Micro ribonucleic acid
MR	Mineralocorticoid receptor
MRA	Mineralocorticoid receptor antagonist
MYBPC3	Myosin-binding protein C
NO–sGC	Nitric oxide soluble guanylate cyclase
PAH	Pulmonary arterial hypertension
PH	Pulmonary hypertension
PH-LHD	Pulmonary hypertension due to left heart disease.
PDE5	Phosphodiesterase type 5
PDE5i	Phosphodiesterase type 5 inhibitor
PVD	Pulmonary vascular disease
RAS	Renin–angiotensin system
RAAS	Renin–angiotensin–aldosterone system
RCT	Randomized clinical trial
RNAi	RNA interference
RV	Right ventricular
SERCA2a	Sarcoplasmic/endoplasmic reticulum Ca^2+^ ATPase 2a
*si*RNA	Small interfering RNA
sGC	Soluble guanylate cyclase
SGLT2i	Sodium–glucose cotransporter 2 inhibitors
SGLT2	Sodium–glucose cotransporter 2
SNS	Sympathetic nervous system
SRX	Super-relaxed state
SVT	Supraventricular tachycardia
TASK1	TWIK-related acid-sensitive potassium channel 1
TGF	Transforming growth factor
TK	Tyrosine kinase
TWIK	Tandem of P-domains in a weakly inward rectifying K^+^ channel
VT	Ventricular tachycardia
WHO	World Health Organization
WPWS	Wolff–Parkinson–White syndrome
